# Endocrine Disorders Are Prominent Clinical Features in Patients With Primary Antibody Deficiencies

**DOI:** 10.3389/fimmu.2019.02079

**Published:** 2019-08-30

**Authors:** Eva C. Coopmans, Paweena Chunharojrith, Sebastian J. C. M. M. Neggers, Marianne W. van der Ent, Sigrid M. A. Swagemakers, Iris H. Hollink, Barbara H. Barendregt, Peter J. van der Spek, Aart-Jan van der Lely, P. Martin van Hagen, Virgil A. S. H. Dalm

**Affiliations:** ^1^Endocrinology Section, Department of Internal Medicine, Pituitary Centre Rotterdam, Erasmus University Medical Centre, Rotterdam, Netherlands; ^2^Academic Center for Rare Immunological Diseases (RIDC), Erasmus MC, University Medical Centre Rotterdam, Rotterdam, Netherlands; ^3^Division of Clinical Immunology, Department of Internal Medicine, Erasmus University Medical Centre, Rotterdam, Netherlands; ^4^Department of Endocrinology, Mahidol University, Bangkok, Thailand; ^5^Department of Pathology and Clinical Bioinformatics, Erasmus University Medical Centre, Rotterdam, Netherlands; ^6^Department of Clinical Genetics, Erasmus University Medical Centre, Rotterdam, Netherlands; ^7^Department of Immunology, Erasmus University Medical Centre, Rotterdam, Netherlands

**Keywords:** common variable immunodefiencies, endocrine disorders, immunodeficiency, endocrine dysfunction, hormones

## Abstract

**Background:** Primary antibody deficiencies (PADs) and anterior pituitary dysfunction are both rare conditions. However, recent studies have remarkably reported the occurrence of anterior pituitary dysfunction in PAD patients.

**Methods:** In this cross-sectional, single-center study we evaluated the prevalence of endocrine disorders in adult PAD patients. Our study focused on common variable immunodeficiency (CVID), immunoglobulin G (IgG) subclass deficiency (IgGSD), and specific anti-polysaccharide antibody deficiency (SPAD). We assessed hormone levels, performed provocative tests and genetic testing in a subset of patients by direct sequencing of the nuclear factor kappa beta subunit 2 (*NFKB2*) gene and primary immunodeficiency (PID) gene panel testing by whole exome sequencing (WES).

**Results:** Our results demonstrated that one out of 24 IgGSD/SPAD patients had secondary hypothyroidism and three out of 9 men with IgGSD/SPAD had secondary hypogonadism. Premature ovarian failure was observed in four out of 9 women with CVID and primary testicular failure in one out of 15 men with CVID. In two out of 26 CVID patients we found partial adrenal insufficiency (AI) and in one out of 18 patients with IgGSD/SPAD secondary AI was found. Moreover, in one out of 23 patients with CVID and in two out of 17 patients with IgGSD/SPAD severe growth hormone deficiency (GHD) was found, while one patient with IgGSD/SPAD showed mild GHD. Combined endocrine disorders were detected in two women with CVID (either partial secondary AI or autoimmune thyroiditis with primary hypogonadism) and in three men with IgGSD/SPAD (two with either mild GHD or secondary hypothyroidism combined with secondary hypogonadism, and one man with secondary AI and severe GHD). Genetic testing in a subset of patients did not reveal pathogenic variants in *NFKB2* or other known PID-associated genes.

**Conclusion:** This is the first study to describe a high prevalence of both anterior pituitary and end-organ endocrine dysfunction in adult PAD patients. As these endocrine disorders may cause considerable health burden, assessment of endocrine axes should be considered in PAD patients.

## Introduction

Primary antibody deficiencies (PADs) are the most common primary immunodeficiencies and are characterized by a B lymphocyte differentiation defect and an impaired production of antigen-specific antibodies resulting in increased risk of infections (1). PADs represent a heterogeneous spectrum of conditions, including common variable immunodeficiency (CVID), immunoglobulin G (IgG) subclass deficiency (IgGSD) and specific anti-polysaccharide antibody deficiency (SPAD).

CVID is a heterogeneous group of disorders primarily characterized by decreased serum IgG and IgA and/or IgM levels and impaired response to immunization (2, 3). CVID is clinically characterized by recurrent and severe infections, polyclonal lymphoproliferation, hematological malignancies, autoimmune diseases, and non-infectious granuloma formation in various organs (2, 3). IgGSD is defined by a reduction in one or more IgG subclasses (1–4), that may result in infectious diseases as well because of poor antibody responses (2, 3). SPAD is characterized by a reduced ability to produce antibodies against polysaccharide antigens (2, 3). Monogenetic defects responsible for CVID have been described in 2–10% of patients, while the genetic defects for IgGSD/SPAD remain unknown to date (3, 4).

Over the past years it has become clear that other, non-immunological comorbidities may occur in PAD patients. Although endocrine disorders are not regularly observed and reported in patients with PAD, recent studies suggest that anterior pituitary dysfunction is more common in patients with PAD than generally assumed.

Anterior pituitary dysfunction, with an estimated prevalence of 1:2,000, is the partial or complete defect in anterior pituitary hormone secretion and may result from pituitary or hypothalamic diseases (5). In 1991, Tovo et al. (6) reported the first case of a patient with CVID and the presence of an isolated adrenocorticotropic hormone (ACTH) deficiency, resulting in secondary adrenal insufficiency (AI) (dysfunction of the anterior pituitary). Since then, several other cases of CVID and secondary AI have been reported in literature (7–21). Quentien et al. (9) demonstrated isolated ACTH deficiency in four patients with CVID, including two siblings, and defined this disease association as a deficit in anterior pituitary function and variable immune deficiency (DAVID). Meanwhile, several cases have reported additional anterior pituitary and end-organ endocrine dysfunction in CVID patients. In particular, growth hormone deficiency (GHD) due to anterior pituitary dysfunction was repeatedly reported (2 patients with mild and 8 patients with severe GHD) (7, 9, 11, 15, 16, 21–24). Other distinct anterior pituitary defects and/or combined with endocrine end-organ dysfunctions were reported (7, 9, 11, 13, 15, 16, 19, 21, 22, 24, 25). The most important characterized reported cases are summarized in Table 1 (6–11, 14, 15, 17–25).

**Table 1:**
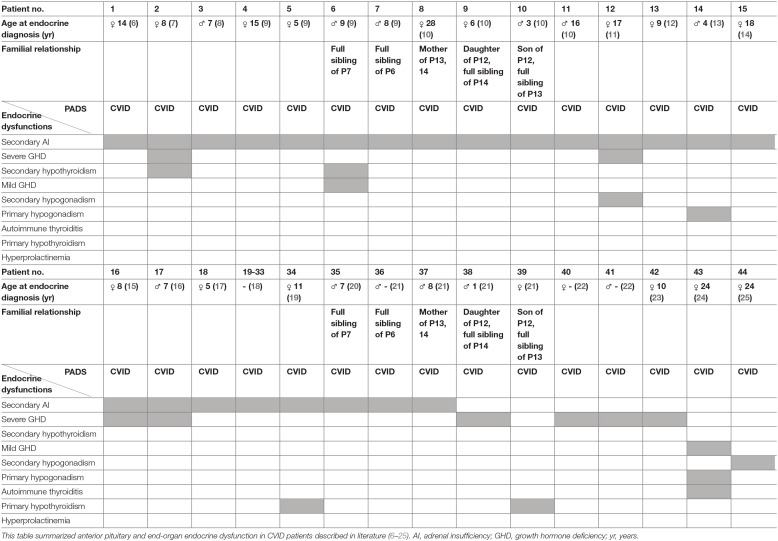
Described endocrine dysfunction in patients with CVID.

*This table summarized anterior pituitary and end-organ endocrine dysfunction in CVID patients described in literature (6–25). AI, adrenal insufficiency; GHD, growth hormone deficiency; yr, years*.

**Figure 1:**
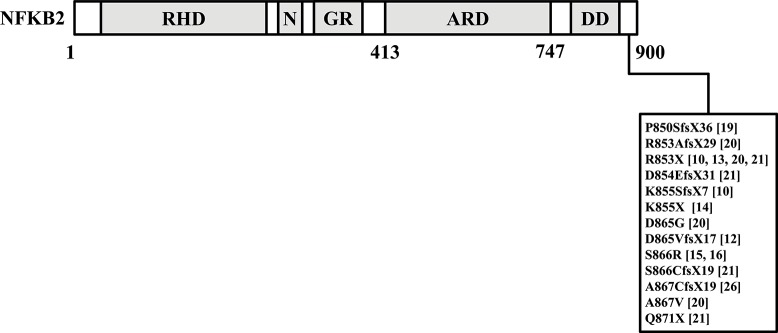
This figure summarized reported germline variants confined to the C-terminal region of the NFKB2 gene in CVID patients with endocrine dysfunctions (10, 12–16, 19–21, 26). NFKB2, nuclear factor kappa beta subunit 2.

Increased insights in causative genetic defects in CVID could potentially explain concomitant endocrine disorders. As an example, Chen et al. (10) reported germline variants near the C-terminal region in nuclear factor kappa beta subunit 2 (*NFKB2*) in CVID patients with secondary AI. Several other groups have also reported variants confined to the C-terminal region of the *NFKB2* gene that could cause combined endocrine- and immunodeficiencies and these are summarized in Figure 1 (10, 12–16, 19–21, 26). It should be stressed that the NFKB signaling has a multitude of diverse functions within the immune system, and the hitherto published phenotypic observations of patients affected by *NFKB2* mutations were highly heterogenic (21).

To our knowledge no studies have attempted to systematically assess the prevalence of endocrine disorders in a cohort of PAD patients. The aim of our study is to investigate the prevalence of anterior pituitary and endocrine end-organ dysfunctions in adult patients with PADs from a tertiary referral center in the Netherlands.

## Methods

### Patients and Ethics

In this single-center cross-sectional study, adult PAD patients were prospectively enrolled between May 2014 and November 2017. All patients (*n*=67) were recruited from the outpatient clinics of the Department of Internal Medicine, Division of Clinical Immunology of the Erasmus University Medical Center, Rotterdam, the Netherlands. All CVID, IgGSD, and SPAD patients fulfilled the International Union of Immunological Societies (IUIS) expert committee diagnostic criteria (27). This study was carried out in accordance with the recommendations of the Medical Ethics Committee of the Erasmus University MC, Rotterdam with written informed consent from all subjects. All subjects gave written informed consent in accordance with the Declaration of Helsinki. The protocol was approved by the Medical Ethics Committee of the Erasmus University MC, Rotterdam (MEC 2013-026, NL40331.078).

### Hormonal Evaluation

Basal concentrations of anterior pituitary and endocrine end-organ hormones were measured including: ACTH, fasting cortisol, free thyroxine (FT4), thyroid-stimulating hormone (TSH), GH, insulin-like growth factor I (IGF-I), prolactin, follicle-stimulating hormone (FSH), luteinizing hormone (LH), in women anti-mullerian hormone (AMH) and estradiol, and in men Inhibin B, sex hormone-binding globulin (SHBG) and testosterone. In patients who were on chronic corticosteroid therapy and who were not able to temporarily discontinue use of corticosteroids for at least 5 days, serum cortisol was not assessed. In all cases with abnormal TSH and/or free thyroxine levels, thyroid autoantibodies (i.e., anti-thyroid peroxidase; anti-TPO, anti-thyroglobulin; anti-Tg and anti-TSH-receptor; anti-TSH-R) were measured.

All blood samples were assessed at the routine Clinical Chemistry Laboratory (AKC) of Erasmus MC. In case patients were on intravenous immunoglobulin (IVIG) treatment, blood samples were collected prior to the IVIG infusion. All samples were collected during an apparent infection-free period, additional serum CRP was measured using Afinion™ Analyzer (Alere Ltd, Stockport, UK) to rule out acute endocrine disorders secondary to current inflammation.

Serum ACTH, cortisol, FSH, LH, prolactin, SHBG and TSH (Immulite 2000 XPi, Siemens AG), GH and IGF-I (IDS-iSYS; Immunodiagnostic Systems Limited, Boldon, UK), estradiol (Cobas e411, Roche Diagnostics GmbH) and free T4 (Vitros ECI, Ortho Clinical Diagnostics, Rochester, NY) were analyzed by automated immunoassays. IGF-I was interpreted according to the sex and age-dependent ranges as defined by Bidlingmaier et al. (28). Serum AMH (Beckman AMH gen II) and Inhibin B (Beckman Inhibin B gen II) were manually determined by ELISA. Testosterone was determined by liquid chromatography-tandem mass spectrometry (PerkinElmer CHS™ MSMS Steroids Kit on a Waters Xevo TQS). Furthermore, serum anti-TPO and anti-TSH-R were measured with and in-house ELISA and anti-Tg was assessed using the ImmunoCAP method (Phadia 250, Uppsala, Sweden).

### Provocative Testing

Provocative testing was performed at our inpatient clinic on a separate occasion when AI and/or GHD was suspected or when patients had another anterior pituitary deficiency. AI was suspected in patients with a fasting basal serum cortisol <266 nmol/L. In patients with basal serum cortisol 266–550 nmol/L, AI is not excluded. Therefore, basal serum cortisol was repeated in these cases and when a second result was still below 550 nmol/L, based on our assay-specific normative data, a provocative test was performed. GHD is suspected in patients when IGF-I levels were below −1 SDs according to Bidlingmaier et al. (28).

Insulin tolerance test (ITT) was used for the assessment of AI or GHD. The ITT was carried out before breakfast after overnight fasting. Venous blood samples were drawn for assessment of cortisol, ACTH, GH, and glucose before (−15 min), and at 0, 20, 30, 40, 60, and 90 min after administration of i.v. insulin. In patients in whom an ITT was contraindicated (i.e., history of coronary artery disease, seizures or stroke; age > 65 is a relative contraindication), an overnight metyrapone test was performed. The test starts at day 1 at 8 a.m. with oral administration of 750 mg of metyrapone, followed by 6 doses additional administration every 4 h. Serum cortisol and 11-deoxycortisol were measured as described above at 8 a.m. at day 2. Growth hormone releasing hormone (GHRH)-arginine test was only performed in patients with isolated decreased IGF-I levels or when ITT results remained inconclusive. The GHRH-arginine test starts by administration of i.v. GHRH (1 μg GHRH/kg bodyweight; BW), followed by a 30 min continuous infusion of 0.5 g arginine/kg BW followed by measurement of GH at −15, 0, 5, 10, 15, 20, 30, 45, 60, 75 and 90 min. In patients who were not able to temporarily discontinue use of corticosteroids for at least 5 days, provocative testing was not performed. The biochemical tests and diagnostic methods were performed in a specialized accredited laboratory for endocrine diseases and confirmed by a senior neuro-endocrinologist (S.N.).

### Neuroradiological Imaging

Magnetic resonance imaging (MRI) of the pituitary was performed according to standard operation procedures of the department of neuroradiology of the Erasmus MC.

### Genetic Analysis

Direct Sanger sequencing of the two C-terminal coding exons of the *NFKB2* gene was performed in a selected group of PAD patients with endocrine dysfunction (*n*=16). We focused on the *NFKB2* gene expression based on previous reports (10, 12–16, 19–21, 26) and its known function in both the immune and endocrine systems. DNA was extracted from peripheral blood samples using standard protocols. *NFKB2* exon 22 and 23 were PCR-amplified with TaqGold™ (Life Technologies) followed by direct sequencing on an ABI Prism 3130 XL fluorescent sequencer (Applied Biosystems, The Netherlands). Sequences were analyzed with CLC DNA workbench software (CLCBio, Aarhus, Denmark) and compared to the NCBI reference sequence (NG_033874).

Additionally, in eight patients PID gene panel testing comprising over 250 PID-associated genes [range 274–367; based on the IUIS classifications 2015 (29) and 2017 (3) was performed using whole exome sequencing (WES). DNA was enriched for the exome using the Agilent Sureselect Clinical Research Exome V2 Capture Enrichment kit (Agilent Technologies) and paired-end sequenced on the Illumina Hiseq platform (GenomeScan, Leiden, the Netherlands). Using our sequencing protocols, the average coverage of the exome is ~50X. Reads were mapped to the genome with the BWA-MEM algorithm (http://bio-bwa.sourceforge.net/) and variant calling was performed by the Genome Analysis Toolkit HaplotypeCaller (http://www.broadinstitute.org/gatk/). Detected variants in the PID-associated genes were filtered and annotated with the Cartagenia software package and classified with Alamut Visual. Detailed information for each panel is listed in Table S1.

### Statistical Analysis

Statistical analyses were performed using SPSS software (version 21 for Windows; SPSS Inc., Chicago, Illinois). Descriptive statistics were used to summarize patient characteristics. The non-parametric unpaired two-samples Wilcoxon test, the Pearson chi-square tests or the Fisher's exact test were used to determine the significance of difference between CVID and IgGSD/SPAD patients. We considered *P*-values of < 0.05 to be statistically significant, *P*-values were not corrected for multiple comparisons.

## Results

### Patient Characteristics

The baseline characteristics of CVID and IgGSD/SPAD patients groups are summarized in Table 2. A total of 67 patients were included in our study and are categorized according to the type of PAD; 43 patients with CVID, 16 patients with IgGSD and 8 patients with SPAD. There is no familial relationship between the patients. Thirty-nine patients received IVIG treatment at doses between 20 and 80 g/4 weeks and 22 patients were on subcutaneous Ig (ScIg) treatment at a dose of 19–53 g/4 weeks. Six patients were not treated with Ig replacement therapy. A significant difference in the number of patients receiving Ig replacement therapy vs. patients receiving no Ig replacement therapy was present between the CVID and IgGSD/SPAD group (Table 2). In all patients the serum CRP levels were within normal range according to reference values from the AKC of Erasmus MC.

**Table 2:** Baseline characteristics of included patient groups.

**Demographic variable**	**CVID**	**IgGSD/SPAD**	***p-*value**
**DIAGNOSIS**			
CVID	43 (64.2)		
IgGSD		16 (23.9)	
SPAD		8 (11.9)	
Mean age at evaluation, yr	47 (17.0)	53 (14.5)	0.099
Male	15 (34.9)	9 (37.5)	0.830
Receive Ig substitution	42 (97.7)	19 (79.2)	0.020

*Data are mean (SD) or n (%). Ig, immunoglobulin; yr, years*.

### Endocrine Disorders

All anterior pituitary and end-organ endocrine dysfunction found in our cohort are summarized in Table 3.

**Table 3:**
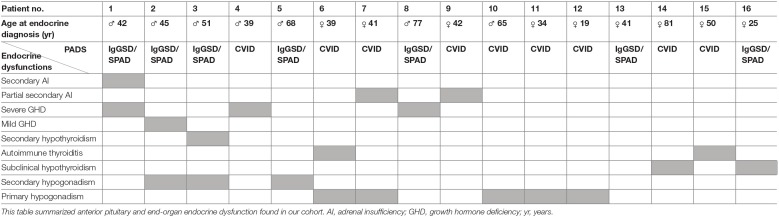
Described endocrine dysfunction in patients with CVID and IgGSD/SPAD.

*This table summarized anterior pituitary and end-organ endocrine dysfunction found in our cohort. AI, adrenal insufficiency; GHD, growth hormone deficiency; yr, years*.

#### Hypothalamic-Pituitary-Gonadal (HPG) Axis

In three out of 9 men with IgGSD/SPAD secondary hypogonadism due to anterior pituitary dysfunction was diagnosed. Male primary gonadal dysfunction was found in one out of 15 men with CVID who presented with primary testicular failure (i.e., low testosterone and high LH and FSH levels) and was treated with testosterone replacement therapy. There were no clinical indications for Klinefelter syndrome or idiopathic granulomatous orchitis, and therefore, we did not obtain karyotype or evaluate for antisperm antibodies.

Primary gonadal dysfunction (i.e., primary hypogonadism) in women was identified in four out of 9 CVID patients (aged≤40 years) that had premature ovarian failure based on low AMH, low estrogen and high LH and FSH levels. Two of these patients were treated with combined oral contraceptive pill. We did not evaluate for adrenal antibodies or thyroid antibodies.

#### Hypothalamic-Pituitary-Thyroid (HPT) Axis

Hypothyroidism with normal TSH levels in one man out of 24 IgGSD/SPAD patients was considered to be due to secondary hypothyroidism (i.e., a dysfunction of the anterior pituitary gland) and was treated with thyroxine replacement therapy.

In four women thyroid dysfunction was found; one woman out of 43 CVID patients demonstrated subclinical hypothyroidism, defined as elevated TSH with normal free thyroxine levels, whereas one of the 24 patients with IgGSD/SPAD (female) had subclinical hypothyroidism including the presence of thyroid antibodies. Autoimmune thyroiditis was identified in two women with CVID; one had Graves' disease and the other had granulomatous thyroiditis without the presence of thyroid antibodies. The granulomatous thyroiditis was detected with somatostatin receptor scintigraphy with ^111^In-pentetreotide (OctreoScan) and confirmed by fine-needle aspiration biopsy of the thyroid gland. However, we cannot rule out that the hypothalamus nor the pituitary gland was affected by granulomatous disease. This area is physiologically positive on OctreoScan. However, patients with granulomatous hypophysitis may benefit from high-dose corticosteroid therapy, which was initiated in this patient when granulomatous thyroiditis was diagnosed.

#### Hypothalamic-Pituitary-Adrenal (HPA) and Somatotropic Axis

Provocative testing was performed when AI or GHD was suspected. ITT was performed in 34 patients, metyrapone test in 9 patients and GHRH-arginine test in 8 patients.

In two out of 26 CVID patients partial secondary AI was observed, where cortisol levels remained below 400–550 nmol/L during ITT. In one of them hydrocortisone replacement therapy was initiated during follow-up due to persistent low cortisol levels. The other patient did not present with symptoms or signs of hypocortisolism but cortisol levels remained suboptimal during follow-up. Therefore, we provided stress instructions and added to the medical record that hydrocortisone replacement therapy was necessary in case of a medical emergency. One out of 18 patients with IgGSD/SPAD secondary AI was observed during ITT and was treated with hydrocortisone replacement therapy.

Severe GHD was observed in one out of 23 patients with CVID and in two out of 17 patients with IgGSD/SPAD. All three patients were treated with GH replacement therapy. Moreover, in one patient in the IgGSD/SPAD group mild GHD was observed.

#### Combined Endocrine Disorders

As was already mentioned above, some patients showed multiple endocrine disorders. Combined anterior pituitary dysfunction was detected in three men with IgGSD/SPAD; one had severe GHD with secondary AI, the second mild GHD and secondary hypogonadism, while the last patient had secondary hypothyroidism and secondary hypogonadism.

In one woman with CVID we detected partial secondary AI and primary hypogonadism, which is a combination of anterior pituitary dysfunction and end-organ dysfunction. Combined endocrine end-organ dysfunction was found in one woman with CVID who presented with granulomatous thyroiditis as well as primary hypogonadism.

MRI scans of the pituitary were performed in the three patients with severe GHD and showed normal pituitary anatomy. It should be noted that pituitary anatomy visualized by MRI is not performed in other patients.

### Genetic Analysis

To evaluate the involvement of *NFKB2* in PAD patients with endocrine dysfunctions, these patients (n=16) were analyzed by sequencing *NFKB2* exon 22 and 23 (Table 3). No pathogenic variant in the C-terminal region of *NFKB2* was detected in any of these patients.

Additionally, 8 out of the 67 PAD patients were investigated for pathogenic variants in PID-associated genes. In one patient (Table 3; no. 1) a heterozygous nuclear factor kappa beta inhibitor alpha (*NFKBIA*)-variant (NM_020529.2(*NFKBIA*):c.554C>T, p.(Thr185Met)) with unknown significance was detected. This variant was not identified in the unaffected father; the mother was unavailable for testing. However, the minor allele frequency in control populations (Genome Aggregation Database v2.1.1; http://gnomad.broadinstitute.org/) was 0,026% (72 out of 282058 alleles) and functional assays in patients cells did not show impairment of *NFKB*-activation and cytokine production (data not shown). No other pathogenic variants or candidates were detected in PID-associated genes in the eight PAD patients.

## Discussion

To our knowledge, this is the first cross-sectional study on the prevalence of endocrine disorders in adult PAD patients. We not only reconfirmed previous findings demonstrating the presence of endocrine disorders of anterior pituitary origin in PAD patients, but we also found end-organ endocrine dysfunction with or without anterior pituitary hormone dysfunction. Moreover, the prevalence of endocrine disorders was unexpectedly high in our patients with CVID, IgGSD, and SPAD.

Our results confirm previous findings that severe GHD is present in CVID patients (7, 11, 15, 21–23). We also observed premature ovarian failure due to end-organ dysfunction in four CVID patients, which was previously described in one case as well (24).

In this study we observed for the first time co-existence of partial secondary AI due to anterior pituitary dysfunction in association with CVID. Moreover, it is also the first time we observed primary testicular failure, subclinical hypothyroidism, granulomatous thyroiditis and Graves' disease due to end-organ dysfunction in CVID patients. Noteworthy is that Graves' disease is a common autoimmune thyroid disease, but since patients with CVID exhibit multiple autoimmune phenomena, we cannot rule out Graves' disease as a comorbidity in CVID. Also, granulomatous complications are a well-known comorbidity of CVID, particularly in the lungs, but these granulomas can be present in all organs, which fits with the presence of granulomatous thyroiditis in one of our CVID patients.

We show for the first time the occurrence of endocrine disorders in adult patients with IgGSD/SPAD. The endocrine disorders of anterior pituitary origin that we observed in the IgGSD/SPAD group were secondary hypogonadism, secondary hypothyroidism, secondary AI and mild and severe GHD. In the latter, MRI scans showed normal pituitary anatomy. Therefore, structural abnormalities (such as an expanding intrasellar mass) or hypophysitis due to autoimmunity or auto-inflammatory conditions are unlikely as causes for GH deficiency. A common genetic cause may explain concomitant GH deficiency in PAD. Moreover, chronic illness is also associated with suppressed GH levels (30) and may account for the high incidence of GH deficiency in PAD patients. Besides, we observed subclinical hypothyroidism due to end-organ dysfunction in the IgGSD/SPAD patients. Although, anterior pituitary hormone deficiencies occurred even more in the IgGSD/SPAD group than in the CVID group, these dysfunctions are not previously described in literature.

PADs and disorders caused by deficiencies in anterior pituitary function are both rare conditions. The high prevalence of endocrine disorders in PAD patients as described in our study may suggests that PADs and anterior pituitary dysfunction are not two distinct phenomena in these patients, but could be both the result of a single pathologic condition.

Previous studies have shown that variant(s) in *NFKB2* (10, 12–16, 19–21, 26) could be considered as underlying genetic defects resulting in both B cell immunodeficiency and endocrine dysfunction. *NFKB2* encodes the full-length p100 protein and serves as central player of the non-canonical *NFKB* signaling pathway, which has a critical role in pituitary development, particularly in differentiation of ACTH-producing corticotroph cells. Interestingly, all of the *NFKB2* variants reported are near the C-terminus of the protein-coding region of *NFKB2*, a region required for the correct processing of the primary translation product (31, 32). It is important to emphasize that the *NFKB2* mutations described so far showed heterogenic clinical expressivity (21) and are associated with a variable penetrance (16), which makes it difficult to predict the phenotype based on the genetic alteration. However, in all our 16 patients with endocrine dysfunctions no pathogenic variants in the C-terminal region of *NFKB2* was detected. Except for the *NFKBIA*-variant of unknown significance in patient no. 1, we did not detect other pathogenic variants in PID-associated genes in eight PAD patients.

We did not identify pathological variation in the coding sequence of the *NFKB2* gene tested, but we believe that the observations described support the existence of a disease association possibly related to a common genetic link. Absence of identification of sequence abnormalities in the open reading frame of the *NFKB2* gene tested might be due to the fact that our study was limited to the coding exons of the gene. Alternatively, it may be explained by the involvement of a number of other currently identified or unknown genes. To elucidate this issue, the possible common molecular cause for combined endocrine- and immunodeficiencies is presently being investigated in our cohort.

Endocrine disorders are not only found in PADs, but are also common features in patients with other primary immunodeficiency diseases (PIDs), such as type 1 diabetes mellitus and thyroiditis in *STAT1* gain-of function (GOF) variant (3), type 1 diabetes mellitus and short statue in *STAT3* GOF variant (33), hypoparathyroidism in DiGeorge syndrome (3) and hypo(para)thyroidism and AI in patients with autoimmune polyendocrinopathy-candidiasis-ectodermal dystrophy (APECED) (3). Future genetic studies may reveal more common genetic pathways involved in immunodeficiency and endocrine dysfunction.

Our report has several limitations. Firstly, we excluded patients who were not able to temporarily discontinue their use of corticosteroids, which interferes with provocative testing. Therefore, the number of patients in which secondary AI was found could be underestimated. Secondly, in our cohort almost all patients were on Ig replacement therapy during the study. The way in which Ig replacement therapy exerts immunomodulatory effects remains unclear, with many pathways, probably mutually non-exclusive, in the innate and adaptive immune systems being potentially targeted. Finally, we focused on the *NFKB2* gene expression only in a selected group of PAD patients with distinctive endocrine dysfunction. Moreover, PID gene panel testing by WES was only performed in eight patients for diagnostic purposes. From these patients, results were included in the current study.

In this study we have observed a remarkably high prevalence of pituitary and end-organ dysfunctions in adult patients with CVID and we expand the phenotype by including IgGSD and SPAD. However, it is not known until now how the immunological and molecular defects in PADs contribute to the development of endocrine disorders. Further genetic studies in PADs may provide new insights into molecular and/or autoimmune mechanisms that could result in these endocrine comorbidities. Endocrine comorbidities in PAD may result in a considerable health burden, we recommend that clinicians consider endocrine disorders in PAD patients and screen for endocrine dysfunction when appropriate.

## Data Availability

The raw data supporting the conclusions of this manuscript will be made available by the authors, without undue reservation, to any qualified researcher.

## Ethics Statement

This study was carried out in accordance with the recommendations of the Medical Ethics Committee of the Erasmus University MC, Rotterdam with written informed consent from all subjects. All subjects gave written informed consent in accordance with the Declaration of Helsinki. The protocol was approved by the Medical Ethics Committee of the Erasmus University MC, Rotterdam (MEC 2013-026, NL40331.078).

## Author Contributions

EC, A-JL, PH, SN, and VD contributed to the study design. EC, PC, and ME collected and provided primary patient data and EC, A-JL, PH, SN, and VD analyzed the clinical results. EC, BB, SS, IH, and PS performed analysis and interpretation of data from genetic testing. EC and VD drafted the manuscript. All other authors were involved in critical revision of the manuscript and approved the final version.

### Conflict of Interest Statement

The authors declare that the research was conducted in the absence of any commercial or financial relationships that could be construed as a potential conflict of interest. The handling editor declared a past co-authorship with the authors VD and PH.
