# Supplementary material for: Endocrine Disorders Are Prominent Clinical Features in Patients With Primary Antibody Deficiencies
Source: Front Immunol. 2019 Aug 30;10:2079. doi: 10.3389/fimmu.2019.02079 (PMC6730260; doi:10.3389/fimmu.2019.02079)
Supplement: Supplementary file 1 [file Data_Sheet_1.PDF]

Whole Exome Sequencing
Gene package Primary Immunodeficiency Disorders, version 2, 1-7-2017

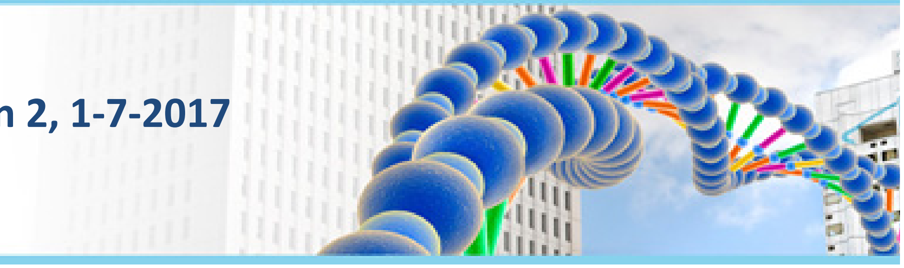

Erasmus MC
University Medical Center Rotterdam

Dept. Clinical Genetics

Technical information

After DNA was enriched using Agilent Sureselect Clinical Research Exome (CRE) Capture, samples were run on the Illumina Hiseq platform. The aim is to obtain 50 million total reads per exome with a mapped fraction >0.98. The average coverage of the exome is ~50x. Data are demultiplexed by Illumina software bcl2fastq. Reads are mapped to the genome using BWA (reference: http://bio-bwa.sourceforge.net/). Variant detection is performed by Genome Analysis Toolkit (reference: http://www.broadinstitute.org/gatk/). Analysis is performed in Cartagenia using The Variant Calling File (VCF) followed by filtering. It is not excluded that pathogenic mutations are being missed using this technology. At this moment, there is not enough information about the sensitivity of this technique with respect to the detection of deletions and duplications of more than 5 nucleotides and of somatic mosaic mutations (all types of sequence changes).

HGNC approved gene symbol	Phenotype description including OMIM phenotype ID(s)	OMIM gene ID	median depth	% covered >10x	% covered >20x
ACD	?Dyskeratosis congenita 6, 616553<br>?Dyskeratosis congenita 7, 616553	609377	Not available		
ACP5	Spondyloenchondrodysplasia with immune dysregulation, 607944	171640			
ACTB	Baraitser-Winter syndrome 1, 243310<br>?Dystonia, juvenile-onset, 607371	102630			
ADA	Adenosine deaminase deficiency, partial, 102700<br>Severe combined immunodeficiency due to ADA deficiency, 102700	608958			
ADA2	Polyarteritis nodosa, childhood-onset, 615688<br>?Sneddon syndrome, 182410	607575			
ADAM17	?Inflammatory skin and bowel disease, neonatal, 1, 614328	603639			
ADAR	Aicardi-Goutieres syndrome 6, 615010<br>Dyschromatosis symmetrica hereditaria, 127400	146920			
AGA	Aspartylglucosaminuria, 208400	613228			
AICDA	Immunodeficiency with hyper-IgM, type 2, 605258	605257			
AIRE	Autoimmune polyendocrinopathy syndrome , type I, with or without reversible metaphyseal dysplasia, 240300	607358			

HGNC approved gene symbol	Phenotype description including OMIM phenotype ID(s)	OMIM gene ID	median depth	% covered >10x	% covered >20x
AK2	Reticular dysgenesis, 267500	103020			
ALG13	?Congenital disorder of glycosylation, type Is, 300884<br>Epileptic encephalopathy, early infantile, 36, 300884	300776			
AP3B1	Hermansky-Pudlak syndrome 2, 608233	603401			
AP4E1	Spastic paraplegia 51, 613744<br>Stuttering, familial persistent, 1, 184450	607244			
APOB	Hypercholesterolemia, due to ligand-defective apo B, 144010<br>Hypobetalipoproteinemia, 615558	107730			
APOL1	{End-stage renal disease, nondiabetic, susceptibility to}, 612551<br>{Glomerulosclerosis, focal segmental, 4, susceptibility to}, 612551	603743			
ARX	Epileptic encephalopathy, early infantile, 1, 308350<br>Hydranencephaly with abnormal genitalia, 300215<br>Lissencephaly 2, 300215<br>Mental retardation 29 and others, 300419<br>Partington syndrome, 309510<br>Proud syndrome, 300004	300382			
ATM	Ataxia-telangiectasia, 208900<br>{Breast cancer, susceptibility to}, 114480<br>Lymphoma, B-cell non-Hodgkin, somatic<br>Lymphoma, mantle cell, somatic<br>T-cell prolymphocytic leukemia, somatic	607585			
ATP2A2	Acrokeratosis verruciformis, 101900<br>Darier disease, 124200	108740			
B2M	?Amyloidosis, familial visceral, 105200<br>Immunodeficiency 43, 241600	109700			
BCL10	?Immunodeficiency 37, 616098<br>Lymphoma, MALT, somatic, 137245<br>{Lymphoma, follicular, somatic}, 605027<br>{Male germ cell tumor, somatic},, 273300<br>{Mesothelioma, somatic}, 156240<br>{Sezary syndrome, somatic}	603517			
BLM	Bloom syndrome, 210900	604610			
BLNK	Agammaglobulinemia 4, 613502	604515			
BLOC1S6	Hermansky-pudlak syndrome 9, 614171	604310			

HGNC approved gene symbol	Phenotype description including OMIM phenotype ID(s)	OMIM gene ID	median depth	% covered >10x	% covered >20x
BTK	Agammaglobulinemia and isolated hormone deficiency, 307200<br>Agammaglobulinemia 1, 300755	300300			
BTLA	No OMIM phenotype	607925			
C1QA	C1q deficiency, 613652	120550			
C1QB	C1q deficiency, 613652	120570			
C1QC	C1q deficiency, 613652	120575			
C1S	C1s deficiency, 613783<br>Ehlers-Danlos syndrome, periodontal type, 2, 617174	120580			
C2	C2 deficiency, 217000<br>{Macular degeneration, age-related, 14, reduced risk of}, 615489	613927			
C3	C3 deficiency, 613779<br>{Hemolytic uremic syndrome, atypical, susceptibility to, 5}, 612925<br>{Macular degeneration, age-related, 9}, 611378	120700			
C4A	[Blood group, Rodgers], 614374<br>C4a deficiency, 614380	120810			
C4B	C4B deficiency, 614379	120820			
C5	C5 deficiency, 609536<br>[Eculizumab, poor response to], 615749	120900			
C6	C6 deficiency, 612446<br>Combined C6/C7 deficiency	217050			
C7	C7 deficiency, 610102	217070			
C8A	C8 deficiency, type I, 613790	120950			
C8B	C8 deficiency, type II, 613789	120960			
C8G	No OMIM phenotype	120930			
C9	C9 deficiency, 613825<br>{Macular degeneration, age-related, 15, susceptibility to}, 615591	120940			
C9orf142	No OMIM phenotype	616315			
CARD11	B-cell expansion with NFKB and T-cell anergy, 616452<br>Immunodeficiency 11, 615206	607210			
CARD14	Pityriasis rubra pilaris, 173200<br>Psoriasis 2, 602723	607211			
CARD9	Candidiasis, familial, 2, 212050	607212			
CARMIL2	No OMIM phenotype	610859			

HGNC approved gene symbol	Phenotype description including OMIM phenotype ID(s)	OMIM gene ID	median depth	% covered >10x	% covered >20x
CASP10	Autoimmune lymphoproliferative syndrome, type II, 603909	601762			
	Gastric cancer, somatic, 613659				
	Lymphoma, non-Hodgkin, somatic, 605027				
CASP8	?Autoimmune lymphoproliferative syndrome, type IIB, 607271	601763			
	{Breast cancer, protection against}, 114480				
	Hepatocellular carcinoma, somatic, 114550				
	{Lung cancer, protection against}, 211980				
CAVIN1	Lipodystrophy, congenital generalized, type 4 , 613327	603198			
CBL	?Juvenile myelomonocytic leukemia, 607785	165360			
	Noonan syndrome-like disorder with or without juvenile myelomonocytic leukemia, 613563				
CCBE1	Hennekam lymphangiectasia-lymphedema syndrome 1, 235510	612753			
CD19	Immunodeficiency, common variable, 3, 613493	107265			
CD247	?Immunodeficiency 25, 610163	186780			
CD27	Lymphoproliferative syndrome 2, 615122	186711			
CD3D	Immunodeficiency 19, 615617	186790			
CD3E	Immunodeficiency 18, 615615	186830			
	Immunodeficiency 18, SCID variant, 615615				
CD3G	Immunodeficiency 17, CD3 gamma deficient, 615607	186740			
CD40	Immunodeficiency with hyper-IgM, type 3, 606843	109535			
CD40LG	Immunodeficiency, with hyper-IgM, 308230	300386			
CD46	{Hemolytic uremic syndrome, atypical, susceptibility to, 2}, 612922	120920			
CD55	[Blood group Cromer], 613793	125240			
CD59	Hemolytic anemia, CD59-mediated, with or without immune-mediated polyneuropathy, 612300	107271			
CD79A	Agammaglobulinemia 3, 613501	112205			
CD79B	Agammaglobulinemia 6, 612692	147245			
CD81	Immunodeficiency, common variable, 6, 613496	186845			
CD8A	CD8 deficiency, familial, 608957	186910			
CDCA7	Immunodeficiency-centromeric instability-facial anomalies syndrome 3, 616910	609937			
CDKN2B	No OMIM phenotype	600431			
CEBPE	Specific granule deficiency, 245480	600749			
CFB	?Complement factor B deficiency, 615561	138470			
	{Hemolytic uremic syndrome, atypical, susceptibility to, 4}, 612924				
	{Macular degeneration, age-related, 14, reduced risk of}, 615489				
CFD	Complement factor D deficiency, 613912	134350			

HGNC approved gene symbol	Phenotype description including OMIM phenotype ID(s)	OMIM gene ID	median depth	% covered >10x	% covered >20x
CFH	Basal laminar drusen, 126700<br>Complement factor H deficiency, 609814<br>{Hemolytic uremic syndrome, atypical, susceptibility to, 1}, 235400<br>{Macular degeneration, age-related, 4}, 610698	134370			
CFHR1	{Hemolytic uremic syndrome, atypical, susceptibility to}, 235400<br>{Macular degeneration, age-related, reduced risk of}, 603075	134371			
CFHR2	No OMIM phenotype	600889			
CFHR3	{Hemolytic uremic syndrome, atypical, susceptibility to}, 235400<br>{Macular degeneration, age-related, reduced risk of}, 603075	605336			
CFHR4	No OMIM phenotype	605337			
CFHR5	Nephropathy due to CFHR5 deficiency, 614809	608593			
CFI	Complement factor I deficiency, 610984<br>{Hemolytic uremic syndrome, atypical, susceptibility to, 3}, 612923<br>{Macular degeneration, age-related, 13, susceptibility to}, 615439	217030			
CFP	Properdin deficiency, 312060	300383			
CFTR	{Bronchiectasis with or without elevated sweat chloride 1, modifier of}, 211400<br>Congenital bilateral absence of vas deferens, 277180<br>Cystic fibrosis, 219700<br>{Hypertrypsinemia, neonatal}<br>{Pancreatitis, idiopathic}, 167800<br>Sweat chloride elevation without CF	602421			
CHD7	CHARGE syndrome, 214800<br>Hypogonadotropic hypogonadism 5 with or without anosmia, 612370	608892			
CIITA	Bare lymphocyte syndrome, type II, complementation group A, 209920<br>{Rheumatoid arthritis, susceptibility to}, 180300	600005			
CLEC4D	No OMIM phenotype	609964			
CLEC7A	{Aspergillosis, susceptibility to}, 614079<br>Candidiasis, familial, 4, 613108	606264			
CLPB	3-methylglutaconic aciduria, type VII, with cataracts, neurologic involvement and neutropenia, 616271	616254			
COLEC11	3MC syndrome 2, 265050	612502			
COPA	{Autoimmune interstitial lung, joint, and kidney disease}, 616414	601924			
CORO1A	Immunodeficiency 8, 615401	605000			
CR2	Immunodeficiency, common variable, 7, 614699<br>{Systemic lupus erythematosus, susceptibility to, 9}, 610927	120650			

HGNC approved gene symbol	Phenotype description including OMIM phenotype ID(s)	OMIM gene ID	median depth	% covered >10x	% covered >20x
CREBBP	Rubinstein-Taybi syndrome 1, 180849	600140			
CSF2RA	Surfactant metabolism dysfunction, pulmonary, 4, 300770	306250			
CSF3R	Neutropenia, severe congenital, 7, 617014	138971			
CTC1	Cerebroretinal microangiopathy with calcifications and cysts, 612199	613129			
CTLA4	Autoimmune lymphoproliferative syndrome, type V, 616100<br>{Celiac disease, susceptibility to, 3}, 609755<br>{Diabetes mellitus, insulin-dependent, 12}, 601388<br>{Hashimoto thyroiditis}, 140300<br>{Systemic lupus erythematosus, susceptibility to}, 152700	123890			
CTPS1	Immunodeficiency 24, 615897	123860			
CTSC	Haim-Munk syndrome, 245010<br>Papillon-Lefevre syndrome, 245000<br>Periodontitis 1, juvenile, 170650	602365			
CXCR4	Myelokathexis, isolated<br>WHIM syndrome, 193670	162643			
CYBA	Chronic granulomatous disease, autosomal, due to deficiency of CYBA, 233690	608508			
CYBB	Chronic granulomatous disease, 306400<br>Immunodeficiency 34, mycobacteriosis, 300645	300481			
DCLRE1B	No OMIM phenotype	609683			
DCLRE1C	Omenn syndrome, 603554<br>Severe combined immunodeficiency, Athabascan type, 602450	605988			
DDX58	Singleton-Merten syndrome 2, 616298	609631			
DGAT1	?Diarrhea 7, 615863	604900			
DHFR	Megaloblastic anemia due to dihydrofolate reductase deficiency, 613839	126060			
DKC1	Dyskeratosis congenita, 305000	300126			
DNASE1	{Systemic lupus erythematosus, susceptibility to}, 152700	125505			
DNMT3B	Immunodeficiency-centromeric instability-facial anomalies syndrome 1, 242860	602900			
DOCK2	Immunodeficiency 40, 616433	603122			
DOCK8	Hyper-IgE recurrent infection syndrome, 243700	611432			
ELANE	Neutropenia, cyclic, 162800<br>Neutropenia, severe congenital 1, 202700	130130			
ELF4	No OMIM phenotype	300775			
EPCAM	Colorectal cancer, hereditary nonpolyposis, type 8, 613244<br>Diarrhea 5, with tufting enteropathy, congenital, 613217	185535			

HGNC approved gene symbol	Phenotype description including OMIM phenotype ID(s)	OMIM gene ID	median depth	% covered >10x	% covered >20x
EPG5	Vici syndrome, 242840	615068			
ERCC2	Cerebrooculofacioskeletal syndrome 2, 610756<br>Trichothiodystrophy 1, photosensitive, 601675<br>Xeroderma pigmentosum, group D, 278730	126340			
ERCC3	Trichothiodystrophy 2, photosensitive, 616390<br>Xeroderma pigmentosum, group B, 610651	133510			
F12	Angioedema, hereditary, type III, 610618<br>Factor XII deficiency, 234000	610619			
FADD	Infections, recurrent, with encephalopathy, hepatic dysfunction, and cardiovasuclar malformations, 613759	602457			
FAS	Autoimmune lymphoproliferative syndrome, type IA, 601859<br>{Autoimmune lymphoproliferative syndrome}, 601859<br>Squamous cell carcinoma, burn scar-related, somatic	134637			
FASLG	Autoimmune lymphoproliferative syndrome, type IB, 601859<br>{Lung cancer, susceptibility to}, 211980	134638			
FCGR1A	[IgG receptor I, phagocytic, familial deficiency of]	146760			
FCGR2A	{Lupus nephritis, susceptibility to}, 152700<br>{Malaria, severe, susceptibility to}, 611162<br>{Pseudomonas aeruginosa, susceptibility to chronic infection by, in cystic fibrosis}, 219700	146790			
FCGR2B	{Malaria, resistance to}, 611162<br>{Systemic lupus erythematosus, susceptibility to}, 152700	604590			
FCGR3A	Immunodeficiency 20, 615707	146740			
FCGR3B	Neutropenia, alloimmune neonatal	610665			
FCGRT	No OMIM phenotype	601437			
FCN3	Immunodeficiency due to ficolin 3 deficiency, 613860	604973			
FERMT3	Leukocyte adhesion deficiency, type III, 612840	607901			
FOXN1	T-cell immunodeficiency, congenital alopecia, and nail dystrophy, 601705	600838			
FOXP3	{Diabetes mellitus, type I, susceptibility to}, 222100<br>Immunodysregulation, polyendocrinopathy, and enteropathy, 304790	300292			
FPR1	No OMIM phenotype	136537			
G6PC	Glycogen storage disease Ia, 232200	613742			
G6PC3	Dursun syndrome, 612541<br>Neutropenia, severe congenital 4, 612541	611045			

HGNC approved gene symbol	Phenotype description including OMIM phenotype ID(s)	OMIM gene ID	median depth	% covered >10x	% covered >20x
G6PD	Favism, 134700<br>Hemolytic anemia due to G6PD deficiency, 300908<br>{Resistance to malaria due to G6PD deficiency}, 611162	305900			
GATA2	Emberger syndrome, 614038<br>Immunodeficiency 21, 614172<br>{Leukemia, acute myeloid, susceptibility to}, 601626<br>{Myelodysplastic syndrome, susceptibility to}, 614286	137295			
GFI1	?Neutropenia, nonimmune chronic idiopathic, of adults, 607847<br>?Neutropenia, severe congenital 2, 613107	600871			
GJC2	Leukodystrophy, hypomyelinating, 2, 608804<br>Lymphedema, hereditary, IC, 613480<br>Spastic paraplegia 44, 613206	608803			
GRHL2	Deafness 28, 608641<br>Ectodermal dysplasia/short stature syndrome, 616029	608576			
GTF2H5	Trichothiodystrophy 3, photosensitive, 616395	608780			
GUCY2C	Diarrhea 6, 614616<br>Meconium ileus, 614665	601330			
HAX1	Neutropenia, severe congenital 3, 610738	605998			
HELLS	Immunodeficiency-centromeric instability-facial anomalies syndrome 4, 616911	603946			
ICOS	Immunodeficiency, common variable, 1, 607594	604558			
IFIH1	Aicardi-Goutieres syndrome 7, 615846<br>Singleton-Merten syndrome 1, 182250	606951			
IFNGR1	{H. pylori infection, susceptibility to}, 600263<br>{Hepatitis B virus infection, susceptibility to}, 610424<br>Immunodeficiency 27A, mycobacteriosis, AR, 209950<br>Immunodeficiency 27B, mycobacteriosis, AD, 615978<br>{Tuberculosis infection, protection against}, 607948<br>{Tuberculosis, susceptibility to}, 607948	107470			
IFNGR2	Immunodeficiency 28, mycobacteriosis, 614889	147569			
IGLL1	Agammaglobulinemia 2, 613500	146770			
IKBKB	Immunodeficiency 15, 615592	603258			

HGNC approved gene symbol	Phenotype description including OMIM phenotype ID(s)	OMIM gene ID	median depth	% covered >10x	% covered >20x
IKBKG	Ectodermal dysplasia, hypohidrotic, with immune deficiency, 300291	300248			
	Ectodermal, dysplasia, anhidrotic, lymphedema and immunodeficiency, 300301				
	Immunodeficiency 33, 300636				
	Immunodeficiency, isolated, 300584				
	Incontinentia pigmenti, 308300				
	Invasive pneumococcal disease, recurrent isolated, 2, 300640				
IKZF1	Immunodeficiency, common variable, 13, 616873	603023			
IL10	{Graft-versus-host disease, protection against}, 614395	124092			
	{HIV-1, susceptibility to}, 609423				
	{Rheumatoid arthritis, progression of}, 180300				
IL10RA	Inflammatory bowel disease 28, early onset, 613148	146933			
IL10RB	{Hepatitis B virus, susceptibility to}, 610424	123889			
	Inflammatory bowel disease 25, early onset, 612567				
IL12B	Immunodeficiency 29, mycobacteriosis, 614890	161561			
IL12RB1	Immunodeficiency 30, 614891	601604			
IL17F	?Candidiasis, familial, 6, 613956	606496			
IL17RA	Immunodeficiency 51, 613953	605461			
IL17RC	Candidiasis, familial, 9, 616445	610925			
IL18	No OMIM phenotype	600953			
IL1RN	{Gastric cancer risk after H. pylori infection}, 137215	147679			
	Interleukin 1 receptor antagonist deficiency, 612852				
	{Microvascular complications of diabetes 4}, 612628				
IL2	Severe combined immunodeficiency due to IL2 deficiency	147680			
IL21	?Immunodeficiency, common variable, 11, 615767	605384			
IL21R	[IgE, elevated level of], 147050	605383			
	Immunodeficiency, primary, IL21R-related, 615207				
IL2RA	{Diabetes, mellitus, insulin-dependent, susceptibility to, 10}, 601942	147730			
	Immunodeficiency 41 with lymphoproliferation and autoimmunity, 606367				
IL2RG	Combined immunodeficiency, moderate, 312863	308380			
	Severe combined immunodeficiency, 300400				
IL36RN	Psoriasis 14, pustular, 614204	605507			
IL7R	Severe combined immunodeficiency, T-cell negative, B-cell/natural killer cell-positive type, 608971	146661			
INO80	No OMIM phenotype	610169			
INPP5D	No OMIM phenotype	601582			

HGNC approved gene symbol	Phenotype description including OMIM phenotype ID(s)	OMIM gene ID	median depth	% covered >10x	% covered >20x
INSR	Diabetes mellitus, insulin-resistant, with acanthosis nigricans, 610549	147670			
	Hyperinsulinemic hypoglycemia, familial, 5, 609968				
	Leprechaunism, 246200				
	Rabson-Mendenhall syndrome, 262190				
IRAK4	IRAK4 deficiency, 607676	606883			
	Invasive pneumococcal disease, recurrent isolated, 1, 610799				
IRF2BP2	No OMIM phenotype	615332			
IRF7	?Immunodeficiency 39, 616345	605047			
IRF8	Immunodeficiency 32A, mycobacteriosis, 614893	601565			
	Immunodeficiency 32B, monocyte and dendritic cell deficiency, 614894				
ISG15	Immunodeficiency 38, 616126	147571			
ITCH	Autoimmune disease, multisystem, with facial dysmorphism, 613385	606409			
ITGAM	{Systemic lupus erythematous, association with susceptibility to, 6}, 609939	120980			
ITGB2	Leukocyte adhesion deficiency, 116920	600065			
ITK	Lymphoproliferative syndrome 1, 613011	186973			
JAGN1	Neutropenia, severe congenital, 6, 616022	616012			
JAK2	{Budd-Chiari syndrome, somatic}, 600800	147796			
	Erythrocytosis, somatic, 133100				
	Leukemia, acute myeloid, somatic, 601626				
	Myelofibrosis, somatic, 254450				
	Polycythemia vera, somatic, 263300				
	Thrombocythemia 3, 614521				
JAK3	SCID, T-negative/B-positive type, 600802	600173			
KMT2D	Kabuki syndrome 1, 147920	602113			
KRAS	Bladder cancer, somatic, 109800	190070			
	Breast cancer, somatic, 114480				
	Cardiofaciocutaneous syndrome 2, 615278				
	Gastric cancer, somatic, 137215				
	Leukemia, acute myeloid, 601626				
	Lung cancer, somatic, 211980				
	Noonan syndrome 3, 609942				
	Pancreatic carcinoma, somatic, 260350				
	RAS-associated autoimmune leukoproliferative disorder, 614470				
	Schimmelpenning-Feuerstein-Mims syndrome, somatic mosaic, 163200				

HGNC approved gene symbol	Phenotype description including OMIM phenotype ID(s)	OMIM gene ID	median depth	% covered >10x	% covered >20x
LAMTOR2	Immunodeficiency due to defect in MAPBP-interacting protein, 610798	610389			
LCK	?Immunodeficiency 22, 615758	153390			
LCT	Lactase deficiency, congenital, 223000	603202			
LIG1	DNA ligase I deficiency	126391			
LIG4	LIG4 syndrome, 606593<br>{Multiple myeloma, resistance to}, 254500	601837			
LPIN2	Majeed syndrome, 609628	605519			
LRBA	Immunodeficiency, common variable, 8, with autoimmunity, 614700	606453			
LRRC8A	?Agammaglobulinemia 5, 613506	608360			
LTBP3	Dental anomalies and short stature, 601216	602090			
LYST	Chediak-Higashi syndrome, 214500	606897			
MAGT1	Immunodeficiency, with magnesium defect, Epstein-Barr virus infection and neoplasia, 300853	300715			
MAL	No OMIM phenotype	188860			
MALT1	Immunodeficiency 12, 615468	604860			
MAN2B1	Mannosidosis, alpha-, types I and II, 248500	609458			
MANBA	Mannosidosis, beta, 248510	609489			
MAP3K14	No OMIM phenotype	604655			
MASP1	3MC syndrome 1, 257920	600521			
MASP2	MASP2 deficiency, 613791	605102			
MBL2	{Chronic infections, due to MBL deficiency}, 614372	154545			
MC2R	Glucocorticoid deficiency, due to ACTH unresponsiveness, 202200	607397			
MCM4	Natural killer cell and glucocorticoid deficiency with DNA repair defect, 609981	602638			
MEFV	Familial Mediterranean fever, AD, 134610<br>Familial Mediterranean fever, AR, 249100	608107			
MGAM	No OMIM phenotype	154360			
MKL1	Megakaryoblastic leukemia, acute	606078			
MPO	{Alzheimer disease, susceptibility to}, 104300<br>{Lung cancer, protection against, in smokers}<br>Myeloperoxidase deficiency, 254600	606989			
MRE11	Ataxia-telangiectasia-like disorder 1, 604391	600814			
MS4A1	Immunodeficiency, common variable, 5, 613495	112210			
MSN	Immunodeficiency 50, 300988	309845			
MTHFD1	{Abruptio placentae, susceptibility to}<br>{Spina bifida, folate-sensitive, susceptibility to}, 601634	172460			

HGNC approved gene symbol	Phenotype description including OMIM phenotype ID(s)	OMIM gene ID	median depth	% covered >10x	% covered >20x
MTTP	Abetalipoproteinemia, 200100<br>{Metabolic syndrome, protection against}, 605552	157147			
MVK	Hyper-IgD syndrome, 260920<br>Mevalonic aciduria, 610377<br>Porokeratosis 3, multiple types, 175900	251170			
MYD88	Macroglobulinemia, Waldenstrom, somatic, 153600<br>Pyogenic bacterial infections, recurrent, due to MYD88 deficiency, 612260	602170			
MYO5B	Microvillus inclusion disease, 251850	606540			
NBN	Aplastic anemia, 609135<br>Leukemia, acute lymphoblastic, 613065<br>Nijmegen breakage syndrome, 251260	602667			
NCF1	Chronic granulomatous disease due to deficiency of NCF-1, 233700	608512			
NCF2	Chronic granulomatous disease due to deficiency of NCF-2, 233710	608515			
NCF4	?Granulomatous disease, chronic, cytochrome b-positive, type III, 613960	601488			
NCSTN	Acne inversa, familial, 1, 142690	605254			
NEUROG3	Diarrhea 4, malabsorptive, congenital, 610370	604882			
NFAT5	No OMIM phenotype	604708			
NFKB1	Immunodeficiency, common variable, 12, 616576	164011			
NFKB2	Immunodeficiency, common variable, 10, 615577	164012			
NFKBIA	Ectodermal dysplasia, anhidrotic, with T-cell immunodeficiency, 612132	164008			
NHEJ1	Severe combined immunodeficiency with microcephaly, growth retardation, and sensitivity to ionizing radiation, 611291	611290			
NHP2	Dyskeratosis congenita 2, 613987	606470			
NKX2-5	Atrial septal defect 7, with or without AV conduction defects, 108900<br>Conotruncal heart malformations, variable, 217095<br>Hypoplastic left heart syndrome 2, 614435<br>Hypothyroidism, congenital nongoitrous, 5, 225250<br>Tetralogy of Fallot, 187500<br>Ventricular septal defect 3, 614432	600584			
NLRC4	Autoinflammation with infantile enterocolitis, 616050<br>?Familial cold autoinflammatory syndrome 4, 616115	606831			
NLRP1	Autoinflammation with arthritis and dyskeratosis, 617388<br>Palmoplantar carcinoma, multiple self-healing, 615255<br>{Vitiligo-associated multiple autoimmune disease susceptibility 1}, 606579	606636			

HGNC approved gene symbol	Phenotype description including OMIM phenotype ID(s)	OMIM gene ID	median depth	% covered >10x	% covered >20x
NLRP12	Familial cold autoinflammatory syndrome 2, 611762	609648			
NLRP3	CINCA syndrome, 607115<br>Familial cold-induced inflammatory syndrome 1, 120100<br>Muckle-Wells syndrome, 191900	606416			
NLRP7	Hydatidiform mole, recurrent, 1, 231090	609661			
NOD2	Blau syndrome, 186580<br>{Inflammatory bowel disease 1, Crohn disease}, 266600<br>{Psoriatic arthritis, susceptibility to}, 607507<br>{Yao syndrome}, 617321	605956			
NOP10	Dyskeratosis congenita 1, 224230	606471			
NRAS	Colorectal cancer, somatic, 114500<br>Epidermal nevus, somatic, 162900<br>Melanocytic nevus syndrome, congenital, somatic, 137550<br>Neurocutaneous melanosis, somatic, 249400<br>Noonan syndrome 6, 613224<br>?RAS-associated autoimmune lymphoproliferative syndrome type IV, somatic, 614470<br>Schimmelpenning-Feuerstein-Mims syndrome, somatic mosaic, 163200<br>Thyroid carcinoma, follicular, somatic, 188470	164790			
NSMCE3	Lung disease, immunodeficiency, and chromosome breakage syndrome, 617241	608243			
ORAI1	Immunodeficiency 9, 612782<br>Myopathy, tubular aggregate, 2, 615883	610277			
OTULIN	Autoinflammation, panniculitis, and dermatosis syndrome, 617099	615712			
PARN	Dyskeratosis congenita 6, 616353<br>Pulmonary fibrosis and/or bone marrow failure, telomere-related, 4, 616371	604212			
PAX5	{Leukemia, acute lymphoblastic, susceptibility to, 3}, 615545	167414			
PBX1	Leukemia, acute pre-B-cell, 176310	176310			
PCCA	Propionicacidemia, 606054	232000			
PCCB	Propionicacidemia, 606054	232050			
PCSK1	Obesity with impaired prohormone processing, 600955<br>{Obesity, susceptibility to, BMIQ12}, 612362	162150			
PEPD	Prolidase deficiency, 170100	613230			
PGM3	Immunodeficiency 23, 615816	172100			
PIGA	Multiple congenital anomalies-hypotonia-seizures syndrome 2, 300868<br>Paroxysmal nocturnal hemoglobinuria, somatic, 300818	311770			

HGNC approved gene symbol	Phenotype description including OMIM phenotype ID(s)	OMIM gene ID	median depth	% covered >10x	% covered >20x
PIK3CD	Immunodeficiency 14, 615513	602839			
PIK3R1	?Agammaglobulinemia 7, 615214<br>Immunodeficiency 36, 616005<br>SHORT syndrome, 269880	171833			
PLCG2	Autoinflammation, antibody deficiency, and immune dysregulation syndrome, 614878<br>Familial cold autoinflammatory syndrome 3, 614468	600220			
PLG	Dysplasminogenemia, 217090<br>Plasminogen deficiency, type I, 217090	173350			
PMM2	Congenital disorder of glycosylation, type Ia, 212065	601785			
PNLIP	?Pancreatic lipase deficiency, 614338	246600			
PNP	Immunodeficiency due to purine nucleoside phosphorylase deficiency, 613179	164050			
POT1	{Glioma susceptibility 9}, 616568<br>{Melanoma, cutaneous malignant, susceptibility to, 10}, 615848	606478			
PRF1	Aplastic anemia, 609135<br>Hemophagocytic lymphohistiocytosis, familial, 2, 603553<br>Lymphoma, non-Hodgkin, 605027	170280			
PRKCD	Autoimmune lymphoproliferative syndrome, type III, 615559	176977			
PRKDC	Immunodeficiency 26, with or without neurologic abnormalities, 615966	600899			
PRPS1	Arts syndrome, 301835<br>Charcot-Marie-Tooth disease recessive, 5, 311070<br>Deafness 1, 304500<br>Gout, PRPS-related, 300661<br>Phosphoribosylpyrophosphate synthetase superactivity, 300661	311850			
PRSS1	Pancreatitis, hereditary, 167800<br>Trypsinogen deficiency, 614044	276000			
PSENEN	Acne inversa, familial, 2, 613736	607632			
PSMB8	Autoinflammation, lipodystrophy, and dermatosis syndrome, 256040	177046			
PSTPIP1	Pyogenic sterile arthritis, pyoderma gangrenosum, and acne, 604416	606347			
PTPN11	LEOPARD syndrome 1, 151100<br>Leukemia, juvenile myelomonocytic, somatic, 607785<br>Metachondromatosis, 156250<br>Noonan syndrome 1, 163950	176876			

HGNC approved gene symbol	Phenotype description including OMIM phenotype ID(s)	OMIM gene ID	median depth	% covered >10x	% covered >20x
PTPN22	{Diabetes, type 1, susceptibility to}, 222100<br>{Rheumatoid arthritis, susceptibility to}, 180300<br>{Systemic lupus erythematosus susceptibility to}, 152700	600716			
PTPRC	{Hepatitic C virus, susceptibility to}, 609532<br>Severe combined immunodeficiency, T cell-negative, B-cell/natural killer-cell positive, 608971	151460			
RAB27A	Griscelli syndrome, type 2, 607624	603868			
RAC2	Neutrophil immunodeficiency syndrome, 608203	602049			
RAG1	Alpha/beta T-cell lymphopenia with gamma/delta T-cell expansion, severe cytomegalovirus infection, and autoimmunity, 609889<br>Combined cellular and humoral immune defects with granulomas, 233650<br>Omenn syndrome, 603554<br>Severe combined immunodeficiency, B cell-negative, 601457	179615			
RAG2	Combined cellular and humoral immune defects with granulomas, 233650<br>Omenn syndrome, 603554<br>Severe combined immunodeficiency, B cell-negative, 601457	179616			
RASGRP2	?Bleeding disorder, platelet-type, 18, 615888	605577			
RBCK1	Polyglucosan body myopathy 1 with or without immunodeficiency, 615895	610924			
RDX	Deafness 24, 611022	179410			
RECQL4	Baller-Gerold syndrome, 218600<br>RAPADILINO syndrome, 266280<br>Rothmund-Thomson syndrome, 268400	603780			
RFX5	Bare lymphocyte syndrome, type II, complementation group C, 209920<br>Bare lymphocyte syndrome, type II, complementation group E, 209920	601863			
RFX6	Mitchell-Riley syndrome, 615710	612659			
RFXANK	MHC class II deficiency, complementation group B, 209920	603200			
RFXAP	Bare lymphocyte syndrome, type II, complementation group D, 209920	601861			
RHOH	No OMIM phenotype	602037			
RMRP	Anauxetic dysplasia 1, 607095<br>Cartilage-hair hypoplasia, 250250<br>Metaphyseal dysplasia without hypotrichosis, 250460	157660			
RNASEH2A	Aicardi-Goutieres syndrome 4, 610333	606034			
RNASEH2B	Aicardi-Goutieres syndrome 2, 610181	610326			
RNASEH2C	Aicardi-Goutieres syndrome 3, 610329	610330			
RNF168	RIDDLE syndrome, 611943	612688			

HGNC approved gene symbol	Phenotype description including OMIM phenotype ID(s)	OMIM gene ID	median depth	% covered >10x	% covered >20x
RNF31	No OMIM phenotype	612487			
RORC	Immunodeficiency 42, 616622	602943			
RPSA	Asplenia, isolated congenital, 271400	150370			
RRAS2	Ovarian carcinoma	600098			
RSPH9	Ciliary dyskinesia, primary, 12, 612650	612648			
RTEL1	Dyskeratosis congenita 4, 615190<br>Dyskeratosis congenita 5, 615190<br>Pulmonary fibrosis and/or bone marrow failure, telomere-related, 3, 616373	608833			
RTL1	No OMIM phenotype	611896			
SAMHD1	Aicardi-Goutieres syndrome 5, 612952<br>?Chilblain lupus 2, 614415	606754			
SAR1B	Chylomicron retention disease, 246700	607690			
SBDS	{Aplastic anemia, susceptibility to}, 609135<br>Shwachman-Diamond syndrome, 260400	607444			
SEMA3E	?CHARGE syndrome, 214800	608166			
SERAC1	3-methylglutaconic aciduria with deafness, encephalopathy, and Leigh-like syndrome, 614739	614725			
SERPING1	Angioedema, hereditary, types I and II, 106100<br>Complement component 4, partial deficiency of, 120790	606860			
SH2B3	Erythrocytosis, somatic, 133100<br>Myelofibrosis, somatic, 254450<br>Thrombocythemia, somatic, 187950	605093			
SH2D1A	Lymphoproliferative syndrome, 1, 308240	300490			
SH3BP2	Cherubism, 118400	602104			
SI	Sucrase-isomaltase deficiency, congenital, 222900	609845			
SKIV2L	Trichohepatoenteric syndrome 2, 614602	600478			
SLC10A2	Bile acid malabsorption, primary, 613291	601295			
SLC26A3	Diarrhea 1, secretory chloride, congenital, 214700	126650			
SLC29A3	Histiocytosis-lymphadenopathy plus syndrome, 602782	612373			
SLC2A2	{Diabetes mellitus, noninsulin-dependent}, 125853<br>Fanconi-Bickel syndrome, 227810	138160			
SLC2A5	No OMIM phenotype	138230			
SLC35A1	Congenital disorder of glycosylation, type IIf, 603585	605634			
SLC35C1	Congenital disorder of glycosylation, type IIc, 266265	605881			

HGNC approved gene symbol	Phenotype description including OMIM phenotype ID(s)	OMIM gene ID	median depth	% covered >10x	% covered >20x
SLC37A4	Glycogen storage disease Ib, 232220	602671			
	Glycogen storage disease Ic, 232240				
SLC39A4	Acrodermatitis enteropathica, 201100	607059			
SLC46A1	Folate malabsorption, hereditary, 229050	611672			
SLC5A1	Glucose/galactose malabsorption, 606824	182380			
SLC7A7	Lysinuric protein intolerance, 222700	603593			
SMARCAL1	Schimke immunoosseous dysplasia, 242900	606622			
SOCS4	No OMIM phenotype	616337			
SP110	Hepatic venoocclusive disease with immunodeficiency, 235550	604457			
	{Mycobacterium tuberculosis, susceptibility to}, 607948				
SPINK5	Atopy, 147050	605010			
	Netherton syndrome, 256500				
SPINT2	Diarrhea 3, secretory sodium, congenital, syndromic, 270420	605124			
STAT1	Immunodeficiency 31A, mycobacteriosis, 614892	600555			
	Immunodeficiency 31B, mycobacterial and viral infections, 613796				
	Immunodeficiency 31C, 614162				
STAT2	Immunodeficiency 44, 616636	600556			
STAT3	Autoimmune disease, multisystem, infantile-onset, 1, 615952	102582			
	Hyper-IgE recurrent infection syndrome, 147060				
STAT4	{Systemic lupus erythematosus, susceptibility to, 11}, 612253	600558			
STAT5B	Growth hormone insensitivity with immunodeficiency, 245590	604260			
	Leukemia, acute promyelocytic, somatic, 102578				
STAT6	No OMIM phenotype	601512			
STIM1	Immunodeficiency 10, 612783	605921			
	Myopathy, tubular aggregate, 1, 160565				
	Stormorken syndrome, 185070				
STK4	T-cell immunodeficiency, recurrent infections, autoimmunity, and cardiac malformations, 614868	604965			
STX11	Hemophagocytic lymphohistiocytosis, familial, 4, 603552	605014			
STX3	No OMIM phenotype	600876			
STXBP2	Hemophagocytic lymphohistiocytosis, familial, 5, 613101	601717			
TAP1	Bare lymphocyte syndrome, type I, 604571	170260			
TAP2	Bare lymphocyte syndrome, type I, due to TAP2 deficiency, 604571	170261			
	Wegener-like granulomatosis				
TAPBP	Bare lymphocyte syndrome, type I, 604571	601962			

HGNC approved gene symbol	Phenotype description including OMIM phenotype ID(s)	OMIM gene ID	median depth	% covered >10x	% covered >20x
TAZ	Barth syndrome, 302060	300394			
TBK1	Frontotemporal dementia and/or amyotrophic lateral sclerosis 4, 616439	604834			
TBX1	Conotruncal anomaly face syndrome, 217095<br>DiGeorge syndrome, 188400<br>Tetralogy of Fallot, 187500<br>Velocardiofacial syndrome, 192430	602054			
TCF3	Agammaglobulinemia 8, 616941	147141			
TCIRG1	Osteopetrosis 1, 259700	604592			
TCN2	Transcobalamin II deficiency, 275350	613441			
TERC	{Aplastic anemia}, 614743<br>Dyskeratosis congenita 1, 127550<br>{Pulmonary fibrosis, idiopathic, susceptibility to}, 614743	602322			
TERT	{Dyskeratosis congenita 2}, 613989<br>{Dyskeratosis congenita 4}, 613989<br>{Leukemia, acute myeloid}, 601626<br>{Melanoma, cutaneous malignant, 9}, 615134<br>{Pulmonary fibrosis and/or bone marrow failure, telomere-related, 1}, 614742	187270			
TFRC	Immunodeficiency 46, 616740	190010			
THBD	{Hemolytic uremic syndrome, atypical, susceptibility to, 6}, 612926<br>Thrombophilia due to thrombomodulin defect, 614486	188040			
TICAM1	{Herpes simplex encephalitic, susceptibility to, 6}, 614850	607601			
TINF2	Dyskeratosis congenita 3, 613990<br>Revesz syndrome, 268130	604319			
TIRAP	{Bacteremia, protection against}, 614382<br>{Malaria, protection against}, 611162<br>{Pneumococcal disease, invasive, protection against}, 610799<br>{Tuberculosis, protection against}, 607948	606252			
TLR3	{HIV1 infection, resistance to}, 609423<br>{Herpes simplex encephalitis, susceptibility to, 2}, 613002	603029			
TLR4	No OMIM phenotype	603030			
TMC6	Epidermodysplasia verruciformis, 226400	605828			
TMC8	Epidermodysplasia verruciformis, 226400	605829			
TMEM173	STING-associated vasculopathy, infantile-onset, 615934	612374			
TMPRSS15	Enterokinase deficiency, 226200	606635			

HGNC approved gene symbol	Phenotype description including OMIM phenotype ID(s)	OMIM gene ID	median depth	% covered >10x	% covered >20x
TNFAIP3	Autoinflammatory syndrome, familial, Behcet-like, 616744	191163			
TNFRSF11A	Osteolysis, familial expansile, 174810<br>Osteopetrosis 7, 612301<br>{Paget disease of bone 2, early-onset}, 602080	603499			
TNFRSF13B	Immunodeficiency, common variable, 2, 240500<br>Immunoglobulin A deficiency 2, 609529	604907			
TNFRSF13C	Immunodeficiency, common variable, 4, 613494	606269			
TNFRSF1A	{Multiple sclerosis, susceptibility to, 5}, 614810<br>Periodic fever, familial, 142680	191190			
TNFRSF4	?Immunodeficiency 16, 615593	600315			
TNFSF12	No OMIM phenotype	602695			
TNFSF13	No OMIM phenotype	604472			
TNFSF13B	No OMIM phenotype	603969			
TPP1	Ceroid lipofuscinosis, neuronal, 2, 204500<br>Spinocerebellar ataxia 7, 609270	607998			
TPP2	No OMIM phenotype	190470			
TRAF3	{?Herpes simplex encephalitis, susceptibility to, 3}, 614849	601896			
TRAF3IP2	?Candidiasis, familial, 8, 615527<br>{Psoriasis susceptibility 13}, 614070	607043			
TREX1	Aicardi-Goutieres syndrome 1, dominant and recessive, 225750<br>Chilblain lupus, 610448<br>{Systemic lupus erythematosus, susceptibility to}, 152700<br>Vasculopathy, retinal, with cerebral leukodystrophy, 192315	606609			
TRNT1	Retinitis pigmentosa and erythrocytic microcytosis, 616959<br>Sideroblastic anemia with B-cell immunodeficiency, periodic fevers, and developmental delay, 616084	612907			
TTC37	Trichohepatoenteric syndrome 1, 222470	614589			
TTC7A	Gastrointestinal defects and immunodeficiency syndrome, 243150	609332			
TYK2	Immunodeficiency 35, 611521	176941			
UNC119	?Cone-rod dystrophy<br>?Immunodeficiency 13, 615518	604011			
UNC13D	Hemophagocytic lymphohistiocytosis, familial, 3, 608898	608897			
UNC93B1	{Herpes simplex encephalitis, susceptibility to, 1}, 610551	608204			
UNG	Immunodeficiency with hyper IgM, type 5, 608106	191525			
USB1	Poikiloderma with neutropenia, 604173	613276			

HGNC approved gene symbol	Phenotype description including OMIM phenotype ID(s)	OMIM gene ID	median depth	% covered >10x	% covered >20x
VPS13B	Cohen syndrome, 216550	607817			
VPS45	Neutropenia, severe congenital, 5, 615285	610035			
WAS	Neutropenia, severe congenital, 300299<br>Thrombocytopenia, 313900<br>Thrombocytopenia, intermittent, 313900<br>Wiskott-Aldrich syndrome, 301000	300392			
WIPF1	?Wiskott-Aldrich syndrome 2, 614493	602357			
WRAP53	Dyskeratosis congenita 3, 613988	612661			
XBP1	{Major affective disorder-7, susceptibility to}, 612371	194355			
XIAP	Lymphoproliferative syndrome, 2, 300635	300079			
XRCC4	Short stature, microcephaly, and endocrine dysfunction, 616541	194363			
XRCC5	No OMIM phenotype	194364			
XRCC6	No OMIM phenotype	152690			
ZAP70	Autoimmune disease, multisystem, infantile-onset, 2, 617006<br>Immunodeficiency 48, 269840	176947			
ZBTB24	Immunodeficiency-centromeric instability-facial anomalies syndrome-2, 614069	614064			

- Gene symbols according HGCN
- OMIM release used: 2-6-2017
- "No OMIM phenotypes" indicates a gene without a current OMIM association
- OMIM phenotypes between "[ ]", indicate "nondiseases," mainly genetic variations that lead to apparently abnormal laboratory test values
- OMIM phenotypes between "{}", indicate risk factors
- OMIM phenotypes with a question mark, "?", before the disease name indicates an unconfirmed or possibly spurious mapping.
- The statistics above are based on a set of 50 samples
- Median depth is the median of the mean sequence depth over the protein coding exons of the transcript
- % Covered 10x describes the percentage of a gene’s coding sequence that is covered at least 10x
- % Covered 20x describes the percentage of a gene’s coding sequence that is covered at least 20x
