# Supplementary material for: Endocrine Disorders Are Prominent Clinical Features in Patients With Primary Antibody Deficiencies
Source: Front Immunol. 2019 Aug 30;10:2079. doi: 10.3389/fimmu.2019.02079 (PMC6730260; doi:10.3389/fimmu.2019.02079)
Supplement: Supplementary file 3 [file Data_Sheet_3.PDF]

Whole Exome Sequencing
Gene package Primary Immunodeficiency Disorders, version 3, 1-2-2018

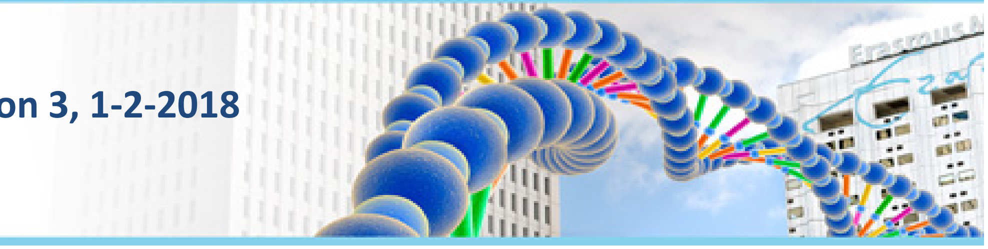

Technical information
DNA was enriched using Agilent SureSelect Clinical Research Exome V2 capture and paired-end sequenced on the Illumina platform (outsourced). The aim is to obtain 8.1 Giga base pairs per exome with a mapped fraction of 0.99. The average coverage of the exome is ~50x. Duplicate reads are excluded. Data are demultiplexed with bcl2fastq Conversion Software from Illumina. Reads are mapped to the genome using the BWA-MEM algorithm (reference: http://bio-bwa.sourceforge.net/). Variant detection is performed by the Genome Analysis Toolkit HaplotypeCaller (reference: http://www.broadinstitute.org/gatk/). The detected variants are filtered and annotated with Cartagenia software and classified with Alamut Visual. It is not excluded that pathogenic mutations are being missed using this technology. At this moment, there is not enough information about the sensitivity of this technique with respect to the detection of deletions and duplications of more than 5 nucleotides and of somatic mosaic mutations (all types of sequence changes).

Erasmus MC
University Medical Center Rotterdam

Dept. Clinical Genetics

HGNC approved gene symbol	Phenotype description including OMIM phenotype ID(s)	OMIM gene ID	median depth	% covered >10x	% covered >20x	% covered 30x
ACD	?Dyskeratosis congenita 6, 616553<br>?Dyskeratosis congenita 7, 616553	609377	92	100	100	100
ACP5	Spondyloenchondrodysplasia with immune dysregulation, 607944	171640	95	100	100	100
ACTB	Baraitser-Winter syndrome 1, 243310<br>?Dystonia, juvenile-onset, 607371	102630	135	100	100	100
ADA	Adenosine deaminase deficiency, partial, 102700<br>Severe combined immunodeficiency due to ADA deficiency, 102700	608958	57	100	98	85
ADA2	Polyarteritis nodosa, childhood-onset, 615688<br>?Sneddon syndrome, 182410	607575	62	100	99	87
ADAM17	?Inflammatory skin and bowel disease, neonatal, 1, 614328	603639	51	100	96	77
ADAR	Aicardi-Goutieres syndrome 6, 615010<br>Dyschromatosis symmetrica hereditaria, 127400	146920	67	100	100	98
AGA	Aspartylglucosaminuria, 208400	613228	53	100	98	85
AICDA	Immunodeficiency with hyper-IgM, type 2, 605258	605257	92	100	100	88
AIRE	Autoimmune polyendocrinopathy syndrome , type I, with or without reversible metaphyseal dysplasia,	607358	55	100	98	83
AK2	Reticular dysgenesis, 267500	103020	43	100	100	83
ALG13	?Congenital disorder of glycosylation, type Is, 300884<br>Epileptic encephalopathy, early infantile, 36, 300884	300776	42	100	96	73

HGNC approved gene symbol	Phenotype description including OMIM phenotype ID(s)	OMIM gene ID	median depth	% covered >10x	% covered >20x	% covered 30x
AP3B1	Hermansky-Pudlak syndrome 2, 608233	603401	50	100	95	71
AP4E1	Spastic paraplegia 51, 613744<br>Stuttering, familial persistent, 1, 184450	607244	50	100	99	87
APOL1	{End-stage renal disease, nondiabetic, susceptibility to}, 612551<br>{Glomerulosclerosis, focal segmental, 4, susceptibility to}, 612551	603743	79	100	100	100
ATM	Ataxia-telangiectasia, 208900<br>{Breast cancer, susceptibility to}, 114480<br>Lymphoma, B-cell non-Hodgkin, somatic<br>Lymphoma, mantle cell, somatic<br>T-cell prolymphocytic leukemia, somatic	607585	55	100	97	83
ATP2A2	Acrokeratosis verruciformis, 101900<br>Darier disease, 124200	108740	67	100	100	98
B2M	?Amyloidosis, familial visceral, 105200<br>Immunodeficiency 43, 241600	109700	62	100	100	100
BCL10	?Immunodeficiency 37, 616098<br>Lymphoma, MALT, somatic, 137245<br>{Lymphoma, follicular, somatic}, 605027<br>{Male germ cell tumor, somatic},, 273300<br>{Mesothelioma, somatic}, 156240<br>{Sezary syndrome, somatic}	603517	57	100	100	89
BLM	Bloom syndrome, 210900	604610	62	100	98	90
BLNK	Agammaglobulinemia 4, 613502	604515	41	100	96	71
BLOC1S6	Hermansky-pudlak syndrome 9, 614171	604310	36	100	92	61
BTK	Agammaglobulinemia and isolated hormone deficiency, 307200<br>Agammaglobulinemia 1, 300755	300300	41	100	91	64
BTLA	No OMIM phenotype	607925	42	100	95	78
C1QA	C1q deficiency, 613652	120550	97	100	100	100
C1QB	C1q deficiency, 613652	120570	85	100	100	96
C1QC	C1q deficiency, 613652	120575	99	100	100	100
C1S	C1s deficiency, 613783<br>Ehlers-Danlos syndrome, periodontal type, 2, 617174	120580	69	100	100	95
C2	C2 deficiency, 217000<br>{Macular degeneration, age-related, 14, reduced risk of}, 615489	613927	74	100	100	98
C3	C3 deficiency, 613779<br>{Hemolytic uremic syndrome, atypical, susceptibility to, 5}, 612925<br>{Macular degeneration, age-related, 9}, 611378	120700	80	100	100	98

HGNC approved gene symbol	Phenotype description including OMIM phenotype ID(s)	OMIM gene ID	median depth	% covered >10x	% covered >20x	% covered 30x
C4A	[Blood group, Rodgers], 614374<br>C4a deficiency, 614380	120810	228	100	100	99
C4B	C4B deficiency, 614379	120820	215	100	100	100
C5	C5 deficiency, 609536<br>[Eculizumab, poor response to], 615749	120900	52	100	97	85
C6	C6 deficiency, 612446<br>Combined C6/C7 deficiency	217050	46	100	97	82
C7	C7 deficiency, 610102	217070	57	100	99	90
C8A	C8 deficiency, type I, 613790	120950	51	100	99	89
C8B	C8 deficiency, type II, 613789	120960	69	100	100	95
C8G	No OMIM phenotype	120930	93	100	100	100
C9	C9 deficiency, 613825<br>{Macular degeneration, age-related, 15, susceptibility to}, 615591	120940	52	100	100	95
C9orf142	No OMIM phenotype	616315	75	100	100	98
CARD11	B-cell expansion with NFKB and T-cell anergy, 616452<br>Immunodeficiency 11, 615206	607210	68	100	99	94
CARD14	Pityriasis rubra pilaris, 173200<br>Psoriasis 2, 602723	607211	71	100	100	98
CARD9	Candidiasis, familial, 2, 212050	607212	67	100	100	98
CARMIL2	No OMIM phenotype	610859	76	98	95	92
CASP10	Autoimmune lymphoproliferative syndrome, type II, 603909<br>Gastric cancer, somatic, 613659<br>Lymphoma, non-Hodgkin, somatic, 605027	601762	53	100	100	90
CASP8	?Autoimmune lymphoproliferative syndrome, type IIB, 607271<br>{Breast cancer, protection against}, 114480<br>Hepatocellular carcinoma, somatic, 114550<br>{Lung cancer, protection against}, 211980	601763	65	100	100	90
CAVIN1	Lipodystrophy, congenital generalized, type 4 , 613327	603198	No coverage data			
CBL	?Juvenile myelomonocytic leukemia, 607785<br>Noonan syndrome-like disorder with or without juvenile myelomonocytic leukemia, 613563	165360	54	100	100	97
CCBE1	Hennekam lymphangiectasia-lymphedema syndrome 1, 235510	612753	58	100	98	90
CD19	Immunodeficiency, common variable, 3, 613493	107265	73	100	100	100
CD247	?Immunodeficiency 25, 610163	186780	50	100	99	85
CD27	Lymphoproliferative syndrome 2, 615122	186711	60	100	100	100
CD3D	Immunodeficiency 19, 615617	186790	61	100	100	97
CD3E	Immunodeficiency 18, 615615<br>Immunodeficiency 18, SCID variant, 615615	186830	73	100	100	99

HGNC approved gene symbol	Phenotype description including OMIM phenotype ID(s)	OMIM gene ID	median depth	% covered >10x	% covered >20x	% covered 30x
CD3G	Immunodeficiency 17, CD3 gamma deficient, 615607	186740	52	100	100	78
CD40	Immunodeficiency with hyper-IgM, type 3, 606843	109535	73	100	100	100
CD40LG	Immunodeficiency, with hyper-IgM, 308230	300386	55	100	100	97
CD46	{Hemolytic uremic syndrome, atypical, susceptibility to, 2}, 612922	120920	65	100	100	90
CD55	[Blood group Cromer], 613793	125240	39	89	82	67
CD59	Hemolytic anemia, CD59-mediated, with or without immune-mediated polyneuropathy, 612300	107271	56	100	100	98
CD79A	Agammaglobulinemia 3, 613501	112205	58	100	98	89
CD79B	Agammaglobulinemia 6, 612692	147245	89	100	100	100
CD81	Immunodeficiency, common variable, 6, 613496	186845	83	100	100	97
CD8A	CD8 deficiency, familial, 608957	186910	65	100	98	91
CDCA7	Immunodeficiency-centromeric instability-facial anomalies syndrome 3, 616910	609937	42	100	95	75
CDKN2B	No OMIM phenotype	600431	53	100	100	100
CEBPE	Specific granule deficiency, 245480	600749	79	100	100	100
CFB	?Complement factor B deficiency, 615561<br>{Hemolytic uremic syndrome, atypical, susceptibility to, 4}, 612924<br>{Macular degeneration, age-related, 14, reduced risk of}, 615489	138470	80	100	100	100
CFD	Complement factor D deficiency, 613912	134350	53	100	97	85
CFH	Basal laminar drusen, 126700<br>Complement factor H deficiency, 609814<br>{Hemolytic uremic syndrome, atypical, susceptibility to, 1}, 235400<br>{Macular degeneration, age-related, 4}, 610698	134370	60	100	99	90
CFHR1	{Hemolytic uremic syndrome, atypical, susceptibility to}, 235400<br>{Macular degeneration, age-related, reduced risk of}, 603075	134371	66	100	98	88
CFHR2	No OMIM phenotype	600889	47	100	100	90
CFHR3	{Hemolytic uremic syndrome, atypical, susceptibility to}, 235400<br>{Macular degeneration, age-related, reduced risk of}, 603075	605336	53	100	93	89
CFHR4	No OMIM phenotype	605337	53	100	94	81
CFHR5	Nephropathy due to CFHR5 deficiency, 614809	608593	59	100	100	91
CFI	Complement factor I deficiency, 610984<br>{Hemolytic uremic syndrome, atypical, susceptibility to, 3}, 612923<br>{Macular degeneration, age-related, 13, susceptibility to}, 615439	217030	50	100	97	82
CFP	Properdin deficiency, 312060	300383	74	100	89	83

HGNC approved gene symbol	Phenotype description including OMIM phenotype ID(s)	OMIM gene ID	median depth	% covered >10x	% covered >20x	% covered 30x
CFTR	{Bronchiectasis with or without elevated sweat chloride 1, modifier of}, 211400<br>Congenital bilateral absence of vas deferens, 277180<br>Cystic fibrosis, 219700<br>{Hypertrypsinemia, neonatal}<br>{Pancreatitis, idiopathic}, 167800<br>Sweat chloride elevation without CF	602421	69	100	99	92
CHD7	CHARGE syndrome, 214800<br>Hypogonadotropic hypogonadism 5 with or without anosmia, 612370	608892	60	100	99	93
CIITA	Bare lymphocyte syndrome, type II, complementation group A, 209920<br>{Rheumatoid arthritis, susceptibility to}, 180300	600005	76	100	100	97
CLEC4D	No OMIM phenotype	609964	40	100	100	74
CLEC7A	{Aspergillosis, susceptibility to}, 614079<br>Candidiasis, familial, 4, 613108	606264	45	100	98	77
CLPB	3-methylglutaconic aciduria, type VII, with cataracts, neurologic involvement and neutropenia, 616271	616254	71	100	100	96
COLEC11	3MC syndrome 2, 265050	612502	84	100	100	100
COPA	{Autoimmune interstitial lung, joint, and kidney disease}, 616414	601924	53	100	100	91
CORO1A	Immunodeficiency 8, 615401	605000	103	100	100	100
CR2	Immunodeficiency, common variable, 7, 614699<br>{Systemic lupus erythematosus, susceptibility to, 9}, 610927	120650	48	100	99	91
CREBBP	Rubinstein-Taybi syndrome 1, 180849	600140	81	100	99	91
CSF2RA	Surfactant metabolism dysfunction, pulmonary, 4, 300770	306250	39	92	78	62
CSF3R	Neutropenia, severe congenital, 7, 617014	138971	79	100	100	100
CTC1	Cerebroretinal microangiopathy with calcifications and cysts, 612199	613129	72	100	100	98
CTLA4	Autoimmune lymphoproliferative syndrome, type V, 616100<br>{Celiac disease, susceptibility to, 3}, 609755<br>{Diabetes mellitus, insulin-dependent, 12}, 601388<br>{Hashimoto thyroiditis}, 140300<br>{Systemic lupus erythematosus, susceptibility to}, 152700	123890	65	100	100	98
CTPS1	Immunodeficiency 24, 615897	123860	48	100	98	83
CTSC	Haim-Munk syndrome, 245010<br>Papillon-Lefevre syndrome, 245000<br>Periodontitis 1, juvenile, 170650	602365	46	100	98	79
CXCR4	Myelokathexis, isolated<br>WHIM syndrome, 193670	162643	61	83	83	83
CYBA	Chronic granulomatous disease, autosomal, due to deficiency of CYBA, 233690	608508	60	99	89	79
CYBB	Chronic granulomatous disease, 306400<br>Immunodeficiency 34, mycobacteriosis, 300645	300481	39	100	95	79

HGNC approved gene symbol	Phenotype description including OMIM phenotype ID(s)	OMIM gene ID	median depth	% covered >10x	% covered >20x	% covered 30x
DCLRE1B	No OMIM phenotype	609683	68	100	99	93
DCLRE1C	Omenn syndrome, 603554<br>Severe combined immunodeficiency, Athabascan type, 602450	605988	45	100	96	81
DDX58	Singleton-Merten syndrome 2, 616298	609631	49	100	98	85
DGAT1	?Diarrhea 7, 615863	604900	87	97	93	88
DHFR	Megaloblastic anemia due to dihydrofolate reductase deficiency, 613839	126060	65	100	100	96
DKC1	Dyskeratosis congenita, 305000	300126	45	100	96	83
DNASE1	{Systemic lupus erythematosus, susceptibility to}, 152700	125505	80	100	100	100
DNMT3B	Immunodeficiency-centromeric instability-facial anomalies syndrome 1, 242860	602900	71	100	99	92
DOCK2	Immunodeficiency 40, 616433	603122	51	100	98	88
DOCK8	Hyper-IgE recurrent infection syndrome, 243700	611432	49	100	94	81
EFNA1	No OMIM phenotype	191164	60	100	100	100
ELANE	Neutropenia, cyclic, 162800<br>Neutropenia, severe congenital 1, 202700	130130	67	100	100	98
ELF4	No OMIM phenotype	300775	58	100	100	97
EPG5	Vici syndrome, 242840	615068	49	100	97	84
ERCC2	Cerebrooculofacioskeletal syndrome 2, 610756<br>Trichothiodystrophy 1, photosensitive, 601675<br>Xeroderma pigmentosum, group D, 278730	126340	75	100	100	99
ERCC3	Trichothiodystrophy 2, photosensitive, 616390<br>Xeroderma pigmentosum, group B, 610651	133510	56	100	99	89
F12	Angioedema, hereditary, type III, 610618<br>Factor XII deficiency, 234000	610619	90	100	100	98
FADD	Infections, recurrent, with encephalopathy, hepatic dysfunction, and cardiovasuclar malformations, 613759	602457	76	100	100	100
FAS	Autoimmune lymphoproliferative syndrome, type IA, 601859<br>{Autoimmune lymphoproliferative syndrome}, 601859<br>Squamous cell carcinoma, burn scar-related, somatic	134637	109	100	99	84
FASLG	Autoimmune lymphoproliferative syndrome, type IB, 601859<br>{Lung cancer, susceptibility to}, 211980	134638	46	100	100	96
FCGR1A	[IgG receptor I, phagocytic, familial deficiency of]	146760	83	100	100	97
FCGR2A	{Lupus nephritis, susceptibility to}, 152700<br>{Malaria, severe, susceptibility to}, 611162<br>{Pseudomonas aeruginosa, susceptibility to chronic infection by, in cystic fibrosis}, 219700	146790	96	100	100	100
FCGR2B	{Malaria, resistance to}, 611162<br>{Systemic lupus erythematosus, susceptibility to}, 152700	604590	134	100	100	100
FCGR3A	Immunodeficiency 20, 615707	146740	162	100	100	100
FCGR3B	Neutropenia, alloimmune neonatal	610665	135	100	100	100

HGNC approved gene symbol	Phenotype description including OMIM phenotype ID(s)	OMIM gene ID	median depth	% covered >10x	% covered >20x	% covered 30x
FCGRT	No OMIM phenotype	601437	69	100	93	88
FCN3	Immunodeficiency due to ficolin 3 deficiency, 613860	604973	67	100	100	94
FERMT3	Leukocyte adhesion deficiency, type III, 612840	607901	76	100	99	95
FOXN1	T-cell immunodeficiency, congenital alopecia, and nail dystrophy, 601705	600838	72	100	100	98
FOXP3	{Diabetes mellitus, type I, susceptibility to}, 222100<br>Immunodysregulation, polyendocrinopathy, and enteropathy, 304790	300292	59	100	100	91
FPR1	No OMIM phenotype	136537	83	100	100	100
G6PC	Glycogen storage disease Ia, 232200	613742	64	100	100	95
G6PC3	Dursun syndrome, 612541<br>Neutropenia, severe congenital 4, 612541	611045	72	100	100	100
G6PD	Favism, 134700<br>Hemolytic anemia due to G6PD deficiency, 300908<br>{Resistance to malaria due to G6PD deficiency}, 611162	305900	76	100	100	98
GATA2	Emberger syndrome, 614038<br>Immunodeficiency 21, 614172<br>{Leukemia, acute myeloid, susceptibility to}, 601626<br>{Myelodysplastic syndrome, susceptibility to}, 614286	137295	86	100	100	100
GFI1	?Neutropenia, nonimmune chronic idiopathic, of adults, 607847<br>?Neutropenia, severe congenital 2, 613107	600871	90	100	100	100
GJC2	Leukodystrophy, hypomyelinating, 2, 608804<br>Lymphedema, hereditary, IC, 613480<br>Spastic paraplegia 44, 613206	608803	52	97	86	75
GRHL2	Deafness 28, 608641<br>Ectodermal dysplasia/short stature syndrome, 616029	608576	56	100	99	87
GTF2H5	Trichothiodystrophy 3, photosensitive, 616395	608780	41	100	100	85
HAX1	Neutropenia, severe congenital 3, 610738	605998	79	100	100	99
HELLS	Immunodeficiency-centromeric instability-facial anomalies syndrome 4, 616911	603946	49	100	94	73
ICOS	Immunodeficiency, common variable, 1, 607594	604558	42	100	100	88
IFIH1	Aicardi-Goutieres syndrome 7, 615846<br>Singleton-Merten syndrome 1, 182250	606951	65	100	99	90
IFNGR1	{H. pylori infection, susceptibility to}, 600263<br>{Hepatitis B virus infection, susceptibility to}, 610424<br>Immunodeficiency 27A, mycobacteriosis, AR, 209950<br>Immunodeficiency 27B, mycobacteriosis, AD, 615978<br>{Tuberculosis infection, protection against}, 607948<br>{Tuberculosis, susceptibility to}, 607948	107470	53	100	98	87
IFNGR2	Immunodeficiency 28, mycobacteriosis, 614889	147569	42	100	91	76

HGNC approved gene symbol	Phenotype description including OMIM phenotype ID(s)	OMIM gene ID	median depth	% covered >10x	% covered >20x	% covered 30x
IGLL1	Agammaglobulinemia 2, 613500	146770	96	100	100	100
IKBKB	Immunodeficiency 15, 615592	603258	54	100	99	91
IKBKG	Ectodermal dysplasia, hypohidrotic, with immune deficiency, 300291<br>Ectodermal, dysplasia, anhidrotic, lymphedema and immunodeficiency, 300301<br>Immunodeficiency 33, 300636<br>Immunodeficiency, isolated, 300584<br>Incontinentia pigmenti, 308300<br>Invasive pneumococcal disease, recurrent isolated, 2, 300640	300248	62	100	100	92
IKZF1	Immunodeficiency, common variable, 13, 616873	603023	78	100	99	94
IL10	{Graft-versus-host disease, protection against}, 614395<br>{HIV-1, susceptibility to}, 609423<br>{Rheumatoid arthritis, progression of}, 180300	124092	81	100	98	86
IL10RA	Inflammatory bowel disease 28, early onset, 613148	146933	73	100	100	96
IL10RB	{Hepatitis B virus, susceptibility to}, 610424<br>Inflammatory bowel disease 25, early onset, 612567	123889	42	100	98	81
IL12B	Immunodeficiency 29, mycobacteriosis, 614890	161561	52	100	99	82
IL12RB1	Immunodeficiency 30, 614891	601604	66	100	100	96
IL17F	?Candidiasis, familial, 6, 613956	606496	57	100	100	100
IL17RA	Immunodeficiency 51, 613953	605461	80	100	100	100
IL17RC	Candidiasis, familial, 9, 616445	610925	73	100	100	97
IL18	No OMIM phenotype	600953	31	100	91	58
IL1RN	{Gastric cancer risk after H. pylori infection}, 137215<br>Interleukin 1 receptor antagonist deficiency, 612852<br>{Microvascular complications of diabetes 4}, 612628	147679	55	100	96	83
IL2	Severe combined immunodeficiency due to IL2 deficiency	147680	31	100	86	49
IL21R	[IgE, elevated level of], 147050<br>Immunodeficiency, primary, IL21R-related, 615207	605383	78	100	100	95
IL2RA	{Diabetes, mellitus, insulin-dependent, susceptibility to, 10}, 601942<br>Immunodeficiency 41 with lymphoproliferation and autoimmunity, 606367	147730	67	100	100	94
IL2RG	Combined immunodeficiency, moderate, 312863<br>Severe combined immunodeficiency, 300400	308380	42	100	99	85
IL36RN	Psoriasis 14, pustular, 614204	605507	53	100	100	98
IL7R	Severe combined immunodeficiency, T-cell negative, B-cell/natural killer cell-positive type, 608971	146661	46	100	100	90
INO80	No OMIM phenotype	610169	53	100	94	76
INPP5D	No OMIM phenotype	601582	62	100	99	91

HGNC approved gene symbol	Phenotype description including OMIM phenotype ID(s)	OMIM gene ID	median depth	% covered >10x	% covered >20x	% covered 30x
INSR	Diabetes mellitus, insulin-resistant, with acanthosis nigricans, 610549<br>Hyperinsulinemic hypoglycemia, familial, 5, 609968<br>Leprechaunism, 246200<br>Rabson-Mendenhall syndrome, 262190	147670	74	100	97	94
IRAK4	IRAK4 deficiency, 607676<br>Invasive pneumococcal disease, recurrent isolated, 1, 610799	606883	53	100	97	77
IRF2BP2	No OMIM phenotype	615332	67	100	100	98
IRF7	?Immunodeficiency 39, 616345	605047	86	100	100	98
IRF8	Immunodeficiency 32A, mycobacteriosis, 614893<br>Immunodeficiency 32B, monocyte and dendritic cell deficiency, 614894	601565	87	100	100	92
ISG15	Immunodeficiency 38, 616126	147571	107	100	100	100
ITCH	Autoimmune disease, multisystem, with facial dysmorphism, 613385	606409	43	96	93	76
ITGAM	{Systemic lupus erythematous, association with susceptibility to, 6}, 609939	120980	77	100	99	95
ITGB2	Leukocyte adhesion deficiency, 116920	600065	77	100	100	99
ITK	Lymphoproliferative syndrome 1, 613011	186973	53	100	98	79
JAGN1	Neutropenia, severe congenital, 6, 616022	616012	65	100	100	100
JAK2	{Budd-Chiari syndrome, somatic}, 600800<br>Erythrocytosis, somatic, 133100<br>Leukemia, acute myeloid, somatic, 601626<br>Myelofibrosis, somatic, 254450<br>Polycythemia vera, somatic, 263300<br>Thrombocythemia 3, 614521	147796	48	100	97	81
JAK3	SCID, T-negative/B-positive type, 600802	600173	64	100	99	96
KMT2D	Kabuki syndrome 1, 147920	602113	97	100	100	99
KRAS	Bladder cancer, somatic, 109800<br>Breast cancer, somatic, 114480<br>Cardiofaciocutaneous syndrome 2, 615278<br>Gastric cancer, somatic, 137215<br>Leukemia, acute myeloid, 601626<br>Lung cancer, somatic, 211980<br>Noonan syndrome 3, 609942<br>Pancreatic carcinoma, somatic, 260350<br>RAS-associated autoimmune leukoproliferative disorder, 614470<br>Schimmelpenning-Feuerstein-Mims syndrome, somatic mosaic, 163200	190070	61	100	97	64
LAMTOR2	Immunodeficiency due to defect in MAPBP-interacting protein, 610798	610389	78	100	100	100
LCK	?Immunodeficiency 22, 615758	153390	98	100	100	100
LIG1	DNA ligase I deficiency	126391	59	100	100	95

HGNC approved gene symbol	Phenotype description including OMIM phenotype ID(s)	OMIM gene ID	median depth	% covered >10x	% covered >20x	% covered 30x
LIG4	LIG4 syndrome, 606593<br>{Multiple myeloma, resistance to}, 254500	601837	62	100	100	100
LPIN2	Majeed syndrome, 609628	605519	53	100	99	88
LRBA	Immunodeficiency, common variable, 8, with autoimmunity, 614700	606453	47	100	97	82
LRRC8A	?Agammaglobulinemia 5, 613506	608360	111	100	100	100
LTBP3	Dental anomalies and short stature, 601216	602090	86	100	99	97
LYST	Chediak-Higashi syndrome, 214500	606897	55	100	99	91
MAGT1	Immunodeficiency, with magnesium defect, Epstein-Barr virus infection and neoplasia, 300853	300715	42	100	99	79
MAL	No OMIM phenotype	188860	57	100	98	80
MALT1	Immunodeficiency 12, 615468	604860	51	99	93	77
MAN2B1	Mannosidosis, alpha-, types I and II, 248500	609458	75	100	100	100
MANBA	Mannosidosis, beta, 248510	609489	59	100	98	87
MAP3K14	No OMIM phenotype	604655	75	100	100	98
MASP1	3MC syndrome 1, 257920	600521	70	100	100	92
MASP2	MASP2 deficiency, 613791	605102	60	100	98	91
MBL2	{Chronic infections, due to MBL deficiency}, 614372	154545	78	100	100	99
MC2R	Glucocorticoid deficiency, due to ACTH unresponsiveness, 202200	607397	90	100	100	100
MCM4	Natural killer cell and glucocorticoid deficiency with DNA repair defect, 609981	602638	56	100	99	91
MEFV	Familial Mediterranean fever, AD, 134610<br>Familial Mediterranean fever, AR, 249100	608107	78	100	100	99
MKL1	Megakaryoblastic leukemia, acute	606078	72	98	95	93
MPO	{Alzheimer disease, susceptibility to}, 104300<br>{Lung cancer, protection against, in smokers}<br>Myeloperoxidase deficiency, 254600	606989	92	100	100	100
MRE11	Ataxia-telangiectasia-like disorder, 604391	600814	43	100	91	72
MS4A1	Immunodeficiency, common variable, 5, 613495	112210	63	100	99	88
MSN	Immunodeficiency 50, 300988	309845	43	97	88	76
MTHFD1	{Abruptio placentae, susceptibility to}<br>{Spina bifida, folate-sensitive, susceptibility to}, 601634	172460	55	100	98	86
MVK	Hyper-IgD syndrome, 260920<br>Mevalonic aciduria, 610377<br>Porokeratosis 3, multiple types, 175900	251170	58	100	100	91
MYD88	Macroglobulinemia, Waldenstrom, somatic, 153600<br>Pyogenic bacterial infections, recurrent, due to MYD88 deficiency, 612260	602170	94	100	100	100
NBN	Aplastic anemia, 609135<br>Leukemia, acute lymphoblastic, 613065<br>Nijmegen breakage syndrome, 251260	602667	52	100	95	76

HGNC approved gene symbol	Phenotype description including OMIM phenotype ID(s)	OMIM gene ID	median depth	% covered >10x	% covered >20x	% covered 30x
NCF1	Chronic granulomatous disease due to deficiency of NCF-1, 233700	608512	98	100	100	98
NCF2	Chronic granulomatous disease due to deficiency of NCF-2, 233710	608515	63	100	100	90
NCF4	?Granulomatous disease, chronic, cytochrome b-positive, type III, 613960	601488	67	100	100	99
NCSTN	Acne inversa, familial, 1, 142690	605254	63	100	100	93
NFAT5	No OMIM phenotype	604708	47	100	97	87
NFKB1	Immunodeficiency, common variable, 12, 616576	164011	40	100	93	74
NFKB2	Immunodeficiency, common variable, 10, 615577	164012	102	100	100	99
NFKBIA	Ectodermal dysplasia, anhidrotic, with T-cell immunodeficiency, 612132	164008	84	100	100	98
NHEJ1	Severe combined immunodeficiency with microcephaly, growth retardation, and sensitivity to ionizing radiation, 611291	611290	40	100	99	80
NHP2	Dyskeratosis congenita 2, 613987	606470	66	100	100	100
NKX2-5	Atrial septal defect 7, with or without AV conduction defects, 108900<br>Conotruncal heart malformations, variable, 217095<br>Hypoplastic left heart syndrome 2, 614435<br>Hypothyroidism, congenital nongoitrous, 5, 225250<br>Tetralogy of Fallot, 187500<br>Ventricular septal defect 3, 614432	600584	84	100	100	100
NLRC4	Autoinflammation with infantile enterocolitis, 616050<br>?Familial cold autoinflammatory syndrome 4, 616115	606831	61	100	99	91
NLRP1	Autoinflammation with arthritis and dyskeratosis, 617388<br>Palmoplantar carcinoma, multiple self-healing, 615255<br>{Vitiligo-associated multiple autoimmune disease susceptibility 1}, 606579	606636	71	100	100	98
NLRP12	Familial cold autoinflammatory syndrome 2, 611762	609648	85	100	100	98
NLRP3	CINCA syndrome, 607115<br>Familial cold-induced inflammatory syndrome 1, 120100<br>Muckle-Wells syndrome, 191900	606416	85	100	100	100
NLRP7	Hydatidiform mole, recurrent, 1, 231090	609661	109	100	100	99
NOD2	Blau syndrome, 186580<br>{Inflammatory bowel disease 1, Crohn disease}, 266600<br>{Psoriatic arthritis, susceptibility to}, 607507<br>{Yao syndrome}, 617321	605956	73	100	99	94
NOP10	Dyskeratosis congenita 1, 224230	606471	121	100	100	100

HGNC approved gene symbol	Phenotype description including OMIM phenotype ID(s)	OMIM gene ID	median depth	% covered >10x	% covered >20x	% covered 30x
NRAS	Colorectal cancer, somatic, 114500<br>Epidermal nevus, somatic, 162900<br>Melanocytic nevus syndrome, congenital, somatic, 137550<br>Neurocutaneous melanosis, somatic, 249400<br>Noonan syndrome 6, 613224<br>?RAS-associated autoimmune lymphoproliferative syndrome type IV, somatic, 614470<br>Schimmelpenning-Feuerstein-Mims syndrome, somatic mosaic, 163200<br>Thyroid carcinoma, follicular, somatic, 188470	164790	42	100	100	91
NSMCE3	Lung disease, immunodeficiency, and chromosome breakage syndrome, 617241	608243	129	100	100	100
ORAI1	Immunodeficiency 9, 612782<br>Myopathy, tubular aggregate, 2, 615883	610277	88	99	99	95
OTULIN	Autoinflammation, panniculitis, and dermatosis syndrome, 617099	615712	49	97	89	78
PARN	Dyskeratosis congenita 6, 616353<br>Pulmonary fibrosis and/or bone marrow failure, telomere-related, 4, 616371	604212	43	100	96	77
PAX5	{Leukemia, acute lymphoblastic, susceptibility to, 3}, 615545	167414	74	100	95	93
PBX1	Leukemia, acute pre-B-cell, 176310	176310	54	100	100	93
PCCA	Propionicacidemia, 606054	232000	50	100	99	85
PCCB	Propionicacidemia, 606054	232050	53	100	96	86
PEPD	Prolidase deficiency, 170100	613230	58	100	98	91
PGM3	Immunodeficiency 23, 615816	172100	59	100	99	89
PIGA	Multiple congenital anomalies-hypotonia-seizures syndrome 2, 300868<br>Paroxysmal nocturnal hemoglobinuria, somatic, 300818	311770	65	100	100	99
PIK3CD	Immunodeficiency 14, 615513	602839	83	100	99	98
PIK3R1	?Agammaglobulinemia 7, 615214<br>Immunodeficiency 36, 616005<br>SHORT syndrome, 269880	171833	59	100	99	89
PLCG2	Autoinflammation, antibody deficiency, and immune dysregulation syndrome, 614878<br>Familial cold autoinflammatory syndrome 3, 614468	600220	70	100	99	92
PLG	Dysplasminogenemia, 217090<br>Plasminogen deficiency, type I, 217090	173350	58	100	100	94
PMM2	Congenital disorder of glycosylation, type Ia, 212065	601785	53	100	100	91
PNP	Immunodeficiency due to purine nucleoside phosphorylase deficiency, 613179	164050	54	100	97	89
POT1	{Glioma susceptibility 9}, 616568<br>{Melanoma, cutaneous malignant, susceptibility to, 10}, 615848	606478	53	100	98	82
PRF1	Aplastic anemia, 609135<br>Hemophagocytic lymphohistiocytosis, familial, 2, 603553<br>Lymphoma, non-Hodgkin, 605027	170280	87	100	100	100

HGNC approved gene symbol	Phenotype description including OMIM phenotype ID(s)	OMIM gene ID	median depth	% covered >10x	% covered >20x	% covered 30x
PRKCD	Autoimmune lymphoproliferative syndrome, type III, 615559	176977	77	100	100	99
PRKDC	Immunodeficiency 26, with or without neurologic abnormalities, 615966	600899	51	100	97	83
PRPS1	Arts syndrome, 301835<br>Charcot-Marie-Tooth disease recessive, 5, 311070<br>Deafness 1, 304500<br>Gout, PRPS-related, 300661<br>Phosphoribosylpyrophosphate synthetase superactivity, 300661	311850	43	100	96	79
PSENEN	Acne inversa, familial, 2, 613736	607632	76	100	100	100
PSMB8	Autoinflammation, lipodystrophy, and dermatosis syndrome, 256040	177046	82	100	100	93
PSTPIP1	Pyogenic sterile arthritis, pyoderma gangrenosum, and acne, 604416	606347	71	100	100	95
PTPN11	LEOPARD syndrome 1, 151100<br>Leukemia, juvenile myelomonocytic, somatic, 607785<br>Metachondromatosis, 156250<br>Noonan syndrome 1, 163950	176876	57	100	96	80
PTPN22	{Diabetes, type 1, susceptibility to}, 222100<br>{Rheumatoid arthritis, susceptibility to}, 180300<br>{Systemic lupus erythematosus susceptibility to}, 152700	600716	41	100	96	74
PTPRC	{Hepatitic C virus, susceptibility to}, 609532<br>Severe combined immunodeficiency, T cell-negative, B-cell/natural killer-cell positive, 608971	151460	53	100	98	85
RAB27A	Griscelli syndrome, type 2, 607624	603868	37	100	94	71
RAC2	Neutrophil immunodeficiency syndrome, 608203	602049	64	100	100	100
RAG1	Alpha/beta T-cell lymphopenia with gamma/delta T-cell expansion, severe cytomegalovirus infection, and autoimmunity, 609889<br>Combined cellular and humoral immune defects with granulomas, 233650<br>Omenn syndrome, 603554<br>Severe combined immunodeficiency, B cell-negative, 601457	179615	75	100	100	100
RAG2	Combined cellular and humoral immune defects with granulomas, 233650<br>Omenn syndrome, 603554<br>Severe combined immunodeficiency, B cell-negative, 601457	179616	61	100	100	100
RASGRP1	No OMIM phenotype	603962	50	100	99	91
RASGRP2	?Bleeding disorder, platelet-type, 18, 615888	605577	59	100	100	99
RBCK1	Polyglucosan body myopathy 1 with or without immunodeficiency, 615895	610924	83	100	99	95
RDX	Deafness 24, 611022	179410	49	100	94	75
RECQL4	Baller-Gerold syndrome, 218600<br>RAPADILINO syndrome, 266280<br>Rothmund-Thomson syndrome, 268400	603780	95	100	100	98

HGNC approved gene symbol	Phenotype description including OMIM phenotype ID(s)	OMIM gene ID	median depth	% covered >10x	% covered >20x	% covered 30x
RFX5	Bare lymphocyte syndrome, type II, complementation group C, 209920<br>Bare lymphocyte syndrome, type II, complementation group E, 209920	601863	72	100	100	95
RFXANK	MHC class II deficiency, complementation group B, 209920	603200	71	100	100	99
RFXAP	Bare lymphocyte syndrome, type II, complementation group D, 209920	601861	129	100	100	100
RHOH	No OMIM phenotype	602037	69	100	100	100
RMRP	Anauxetic dysplasia 1, 607095<br>Cartilage-hair hypoplasia, 250250<br>Metaphyseal dysplasia without hypotrichosis, 250460	157660	No coverage data			
RNASEH2A	Aicardi-Goutieres syndrome 4, 610333	606034	73	100	100	99
RNASEH2B	Aicardi-Goutieres syndrome 2, 610181	610326	48	100	97	81
RNASEH2C	Aicardi-Goutieres syndrome 3, 610329	610330	139	100	100	100
RNF168	RIDDLE syndrome, 611943	612688	70	100	98	90
RNF31	No OMIM phenotype	612487	81	100	100	98
RORC	Immunodeficiency 42, 616622	602943	68	100	100	100
RPSA	Asplenia, isolated congenital, 271400	150370	72	100	100	100
RRAS2	Ovarian carcinoma	600098	48	100	96	77
RSPH9	Ciliary dyskinesia, primary, 12, 612650	612648	85	100	100	99
RTEL1	Dyskeratosis congenita 4, 615190<br>Dyskeratosis congenita 5, 615190<br>Pulmonary fibrosis and/or bone marrow failure, telomere-related, 3, 616373	608833	80	100	100	98
RTL1	No OMIM phenotype	611896	119	100	100	99
SAMHD1	Aicardi-Goutieres syndrome 5, 612952<br>?Chilblain lupus 2, 614415	606754	49	100	93	69
SBDS	{Aplastic anemia, susceptibility to}, 609135<br>Shwachman-Diamond syndrome, 260400	607444	49	100	100	92
SEMA3E	?CHARGE syndrome, 214800	608166	44	100	98	78
SERAC1	3-methylglutaconic aciduria with deafness, encephalopathy, and Leigh-like syndrome, 614739	614725	44	100	92	66
SERPING1	Angioedema, hereditary, types I and II, 106100<br>Complement component 4, partial deficiency of, 120790	606860	74	100	100	96
SH2B3	Erythrocytosis, somatic, 133100<br>Myelofibrosis, somatic, 254450<br>Thrombocythemia, somatic, 187950	605093	76	100	100	100
SH2D1A	Lymphoproliferative syndrome, 1, 308240	300490	44	100	99	79
SH3BP2	Cherubism, 118400	602104	62	91	91	91
SKIV2L	Trichohepatoenteric syndrome 2, 614602	600478	87	100	100	100
SLC29A3	Histiocytosis-lymphadenopathy plus syndrome, 602782	612373	85	100	100	99
SLC35A1	Congenital disorder of glycosylation, type IIf, 603585	605634	49	100	99	82

HGNC approved gene symbol	Phenotype description including OMIM phenotype ID(s)	OMIM gene ID	median depth	% covered >10x	% covered >20x	% covered 30x
SLC35C1	Congenital disorder of glycosylation, type IIc, 266265	605881	89	100	100	99
SLC37A4	Glycogen storage disease Ib, 232220<br>Glycogen storage disease Ic, 232240	602671	69	100	100	97
SLC39A4	Acrodermatitis enteropathica, 201100	607059	71	100	100	100
SLC46A1	Folate malabsorption, hereditary, 229050	611672	75	100	100	99
SMARCAL1	Schimke immunoosseous dysplasia, 242900	606622	61	100	99	93
SOCS4	No OMIM phenotype	616337	64	100	100	100
SP110	Hepatic venoocclusive disease with immunodeficiency, 235550<br>{Mycobacterium tuberculosis, susceptibility to}, 607948	604457	44	100	99	85
SPINK5	Atopy, 147050<br>Netherton syndrome, 256500	605010	46	100	96	82
SRP54	No OMIM phenotype	604857	42	100	96	81
STAT1	Immunodeficiency 31A, mycobacteriosis, 614892<br>Immunodeficiency 31B, mycobacterial and viral infections, 613796<br>Immunodeficiency 31C, 614162	600555	43	100	96	80
STAT2	Immunodeficiency 44, 616636	600556	60	100	99	91
STAT3	Autoimmune disease, multisystem, infantile-onset, 1, 615952<br>Hyper-IgE recurrent infection syndrome, 147060	102582	60	100	100	91
STAT4	{Systemic lupus erythematosus, susceptibility to, 11}, 612253	600558	44	100	95	77
STAT5B	Growth hormone insensitivity with immunodeficiency, 245590<br>Leukemia, acute promyelocytic, somatic, 102578	604260	71	100	99	89
STAT6	No OMIM phenotype	601512	60	100	99	93
STIM1	Immunodeficiency 10, 612783<br>Myopathy, tubular aggregate, 1, 160565<br>Stormorken syndrome, 185070	605921	64	100	100	94
STK4	T-cell immunodeficiency, recurrent infections, autoimmunity, and cardiac malformations, 614868	604965	58	100	100	93
STX11	Hemophagocytic lymphohistiocytosis, familial, 4, 603552	605014	162	100	100	100
STXBP2	Hemophagocytic lymphohistiocytosis, familial, 5, 613101	601717	68	100	100	93
TAP1	Bare lymphocyte syndrome, type I, 604571	170260	83	100	100	99
TAP2	Bare lymphocyte syndrome, type I, due to TAP2 deficiency, 604571<br>Wegener-like granulomatosis	170261	67	100	100	97
TAPBP	Bare lymphocyte syndrome, type I, 604571	601962	70	100	100	100
TAZ	Barth syndrome, 302060	300394	59	100	91	90
TBK1	Frontotemporal dementia and/or amyotrophic lateral sclerosis 4, 616439	604834	57	100	97	82

HGNC approved gene symbol	Phenotype description including OMIM phenotype ID(s)	OMIM gene ID	median depth	% covered >10x	% covered >20x	% covered 30x
TBX1	Conotruncal anomaly face syndrome, 217095<br>DiGeorge syndrome, 188400<br>Tetralogy of Fallot, 187500<br>Velocardiofacial syndrome, 192430	602054	44	91	78	67
TCF3	Agammaglobulinemia 8, 616941	147141	61	100	100	94
TCIRG1	Osteopetrosis 1, 259700	604592	79	100	100	92
TCN2	Transcobalamin II deficiency, 275350	613441	76	100	100	99
TERC	{Aplastic anemia}, 614743<br>Dyskeratosis congenita 1, 127550<br>{Pulmonary fibrosis, idiopathic, susceptibility to}, 614743	602322	No coverage data			
TERT	{Dyskeratosis congenita 2}, 613989<br>{Dyskeratosis congenita 4}, 613989<br>{Leukemia, acute myeloid}, 601626<br>{Melanoma, cutaneous malignant, 9}, 615134<br>{Pulmonary fibrosis and/or bone marrow failure, telomere-related, 1}, 614742	187270	85	100	100	94
TFRC	Immunodeficiency 46, 616740	190010	41	100	96	70
THBD	{Hemolytic uremic syndrome, atypical, susceptibility to, 6}, 612926<br>Thrombophilia due to thrombomodulin defect, 614486	188040	131	100	100	100
TICAM1	{Herpes simplex encephalitic, susceptibility to, 6}, 614850	607601	107	100	100	100
TINF2	Dyskeratosis congenita 3, 613990<br>Revesz syndrome, 268130	604319	94	100	100	100
TIRAP	{Bacteremia, protection against}, 614382<br>{Malaria, protection against}, 611162<br>{Pneumococcal disease, invasive, protection against}, 610799<br>{Tuberculosis, protection against}, 607948	606252	86	100	100	100
TLR3	{HIV1 infection, resistance to}, 609423<br>{Herpes simplex encephalitis, susceptibility to, 2}, 613002	603029	54	100	100	97
TLR4	No OMIM phenotype	603030	49	100	100	97
TMC6	Epidermodysplasia verruciformis, 226400	605828	59	100	100	98
TMC8	Epidermodysplasia verruciformis, 226400	605829	68	100	100	98
TMEM173	STING-associated vasculopathy, infantile-onset, 615934	612374	79	100	100	100
TNFAIP3	Autoinflammatory syndrome, familial, Behcet-like, 616744	191163	66	100	98	90
TNFRSF11A	Osteolysis, familial expansile, 174810<br>Osteopetrosis 7, 612301<br>{Paget disease of bone 2, early-onset}, 602080	603499	66	95	95	91
TNFRSF13B	Immunodeficiency, common variable, 2, 240500<br>Immunoglobulin A deficiency 2, 609529	604907	55	100	100	96

HGNC approved gene symbol	Phenotype description including OMIM phenotype ID(s)	OMIM gene ID	median depth	% covered >10x	% covered >20x	% covered 30x
TNFRSF13C	Immunodeficiency, common variable, 4, 613494	606269	53	100	84	65
TNFRSF1A	{Multiple sclerosis, susceptibility to, 5}, 614810<br>Periodic fever, familial, 142680	191190	66	100	99	93
TNFRSF4	?Immunodeficiency 16, 615593	600315	61	100	94	84
TNFSF12	No OMIM phenotype	602695	58	100	100	93
TPP1	Ceroid lipofuscinosis, neuronal, 2, 204500<br>Spinocerebellar ataxia 7, 609270	607998	70	100	100	99
TPP2	No OMIM phenotype	190470	41	100	93	70
TRAF3	{?Herpes simplex encephalitis, susceptibility to, 3}, 614849	601896	69	100	100	97
TRAF3IP2	?Candidiasis, familial, 8, 615527<br>{Psoriasis susceptibility 13}, 614070	607043	70	100	100	93
TREX1	Aicardi-Goutieres syndrome 1, dominant and recessive, 225750<br>Chilblain lupus, 610448<br>{Systemic lupus erythematosus, susceptibility to}, 152700<br>Vasculopathy, retinal, with cerebral leukodystrophy, 192315	606609	116	100	100	100
TRNT1	Retinitis pigmentosa and erythrocytic microcytosis, 616959<br>Sideroblastic anemia with B-cell immunodeficiency, periodic fevers, and developmental delay, 616084	612907	55	100	97	83
TTC37	Trichohepatoenteric syndrome 1, 222470	614589	43	100	97	80
TTC7A	Gastrointestinal defects and immunodeficiency syndrome, 243150	609332	68	100	99	94
TYK2	Immunodeficiency 35, 611521	176941	83	100	100	100
UNC119	?Cone-rod dystrophy<br>?Immunodeficiency 13, 615518	604011	65	100	100	96
UNC13D	Hemophagocytic lymphohistiocytosis, familial, 3, 608898	608897	70	100	100	100
UNC93B1	{Herpes simplex encephalitis, susceptibility to, 1}, 610551	608204	46	93	76	63
UNG	Immunodeficiency with hyper IgM, type 5, 608106	191525	60	100	100	99
USB1	Poikiloderma with neutropenia, 604173	613276	47	100	99	85
VPS13B	Cohen syndrome, 216550	607817	54	100	98	87
VPS45	Neutropenia, severe congenital, 5, 615285	610035	52	100	99	86
WAS	Neutropenia, severe congenital, 300299<br>Thrombocytopenia, 313900<br>Thrombocytopenia, intermittent, 313900<br>Wiskott-Aldrich syndrome, 301000	300392	54	96	84	75
WIPF1	?Wiskott-Aldrich syndrome 2, 614493	602357	82	100	98	88
WRAP53	Dyskeratosis congenita 3, 613988	612661	95	100	100	100
XBP1	{Major affective disorder-7, susceptibility to}, 612371	194355	51	100	98	89
XIAP	Lymphoproliferative syndrome, 2, 300635	300079	52	100	96	85
XRCC4	Short stature, microcephaly, and endocrine dysfunction, 616541	194363	41	100	97	78

HGNC approved gene symbol	Phenotype description including OMIM phenotype ID(s)	OMIM gene ID	median depth	% covered >10x	% covered >20x	% covered 30x
XRCC5	No OMIM phenotype	194364	52	100	99	88
XRCC6	No OMIM phenotype	152690	110	100	98	90
ZAP70	Autoimmune disease, multisystem, infantile-onset, 2, 617006<br>Immunodeficiency 48, 269840	176947	86	100	100	100
ZBTB24	Immunodeficiency-centromeric instability-facial anomalies syndrome-2, 614069	614064	57	100	100	99

- Gene symbols according HGCN
- OMIM release used: 2-6-2017
- "No OMIM phenotypes" indicates a gene without a current OMIM association
- OMIM phenotypes between "[ ]", indicate "nondiseases," mainly genetic variations that lead to apparently abnormal laboratory test values
- OMIM phenotypes between "{}", indicate risk factors
- OMIM phenotypes with a question mark, "?", before the disease name indicates an unconfirmed or possibly spurious mapping
- The statistics above are based on a set of 96 samples
- Median depth is the median of the mean sequence depth over the protein coding exons (±10bp flanking introns) of the longest transcript
- % Covered 10x describes the percentage of a gene's coding sequence (±10bp flanking introns) that is covered at least 10x
- % Covered 20x describes the percentage of a gene's coding sequence (±10bp flanking introns) that is covered at least 20x
