# Supplementary material for: Endocrine Disorders Are Prominent Clinical Features in Patients With Primary Antibody Deficiencies
Source: Front Immunol. 2019 Aug 30;10:2079. doi: 10.3389/fimmu.2019.02079 (PMC6730260; doi:10.3389/fimmu.2019.02079)
Supplement: Supplementary file 4 [file Data_Sheet_4.PDF]

Whole Exome Sequencing
Gene package Primary Immunodeficiency Disorders, version 4, 30-7-2018

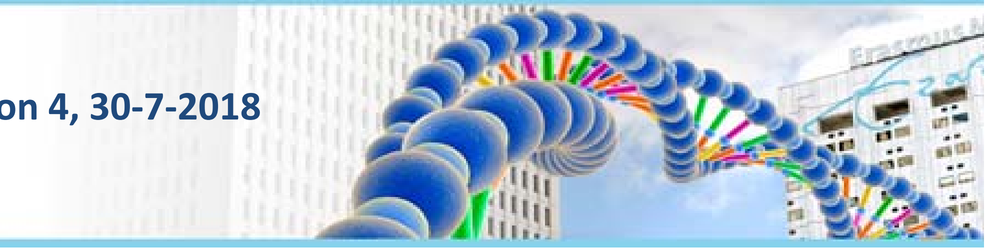

Technical information

DNA was enriched using Agilent SureSelect Clinical Research Exome V2 capture and paired-end sequenced on the Illumina platform (outsourced). The aim is to obtain 8.1 Giga base pairs per exome with a mapped fraction of 0.99. The average coverage of the exome is ~50x. Duplicate reads are excluded. Data are demultiplexed with bcl2fastq Conversion Software from Illumina. Reads are mapped to the genome using the BWA-MEM algorithm (reference: http://bio-bwa.sourceforge.net/). Variant detection is performed by the Genome Analysis Toolkit HaplotypeCaller (reference: http://www.broadinstitute.org/gatk/). The detected variants are filtered and annotated with Cartagenia software and classified with Alamut Visual. It is not excluded that pathogenic mutations are being missed using this technology. At this moment, there is not enough information about the sensitivity of this technique with respect to the detection of deletions and duplications of more than 5 nucleotides and of somatic mosaic mutations (all types of sequence changes).

Erasmus MC
University Medical Center Rotterdam

Dept. Clinical Genetics

HGNC approved gene symbol	Phenotype description including OMIM phenotype ID(s)	OMIM gene ID	median depth	% covered >10x	% covered >20x	% covered 30x
ACD	?Dyskeratosis congenita, autosomal dominant 6, autosomal recessive 7 616553	609377	125	100	100	100
ACP5	Spondyloenchondrodysplasia with immune dysregulation, 607944	171640	133	100	100	100
ACTB	Baraitser-Winter syndrome 1, 243310<br>?Dystonia, juvenile-onset, 607371	102630	208	100	100	100
ADA	Adenosine deaminase deficiency, partial, 102700<br>Severe combined immunodeficiency due to ADA deficiency, 102700	608958	79	100	100	95
ADA2	Polyarteritis nodosa, childhood-onset, 615688<br>?Sneddon syndrome, 182410	607575	91	100	100	97
ADAM17	?Inflammatory skin and bowel disease, neonatal, 1, 614328	603639	58	100	98	89
ADAR	Aicardi-Goutieres syndrome 6, 615010<br>Dyschromatosis symmetrica hereditaria, 127400	146920	79	100	100	98
AGA	Aspartylglucosaminuria, 208400	613228	62	100	99	92
AICDA	Immunodeficiency with hyper-IgM, type 2, 605258	605257	128	100	100	92
AIRE	Autoimmune polyendocrinopathy syndrome , type I, with or without reversible metaphyseal dysplasia,	607358	98	100	100	99
AK2	Reticular dysgenesis, 267500	103020	69	100	100	92
ALG13	?Congenital disorder of glycosylation, type Is, 300884<br>Epileptic encephalopathy, early infantile, 36, 300884	300776	41	100	93	68
AP1S3	{Psoriasis 15, pustular, susceptibility to}, 616106	615781	40	100	99	83

HGNC approved gene symbol	Phenotype description including OMIM phenotype ID(s)	OMIM gene ID	median depth	% covered >10x	% covered >20x	% covered 30x
AP3B1	Hermansky-Pudlak syndrome 2, 608233	603401	57	100	96	80
AP3D1	?Hermansky-Pudlak syndrome 10, 617050	607246	101	99	98	97
APOL1	{End-stage renal disease, nondiabetic, susceptibility to}, 612551<br>{Glomerulosclerosis, focal segmental, 4, susceptibility to}, 612551	603743	93	100	100	100
ARPC1B	Platelet abnormalities with eosinophilia and immune-mediated inflammatory disease, 617718	604223	108	100	100	100
ATM	Ataxia-telangiectasia, 208900<br>{Breast cancer, susceptibility to}, 114480<br>Lymphoma, B-cell non-Hodgkin, somatic<br>Lymphoma, mantle cell, somatic<br>T-cell prolymphocytic leukemia, somatic	607585	64	100	98	90
ATP6AP1	Immunodeficiency 47, 300972	300197	70	100	99	96
B2M	?Amyloidosis, familial visceral, 105200<br>Immunodeficiency 43, 241600	109700	143	100	100	100
BACH2	No OMIM phenotype	605394	104	100	100	100
BCL10	?Immunodeficiency 37, 616098<br>Lymphoma, MALT, somatic, 137245<br>{Lymphoma, follicular, somatic}, 605027<br>{Male germ cell tumor, somatic},, 273300<br>{Mesothelioma, somatic}, 156240<br>{Sezary syndrome, somatic}	603517	57	100	100	91
BCL11B	?Immunodeficiency 49, 617237	606558	106	100	96	92
BLK	Maturity-onset diabetes of the young, type 11, 613375	191305	107	100	100	100
BLM	Bloom syndrome, 210900	604610	71	100	99	92
BLNK	?Agammaglobulinemia 4, 613502	604515	53	100	96	82
BLOC1S6	?Hermansky-pudlak syndrome 9, 614171	604310	41	100	98	75
BTK	Agammaglobulinemia and isolated hormone deficiency, 307200<br>Agammaglobulinemia, X-linked 1, 300755	300300	41	100	96	68
C1QA	C1q deficiency, 613652	120550	134	100	100	100
C1QB	C1q deficiency, 613652	120570	112	100	100	99
C1QC	C1q deficiency, 613652	120575	143	100	100	100
C1S	C1s deficiency, 613783<br>Ehlers-Danlos syndrome, periodontal type, 2, 617174	120580	86	100	100	96
C2	C2 deficiency, 217000<br>{Macular degeneration, age-related, 14, reduced risk of}, 615489	613927	95	100	100	99
C3	C3 deficiency, 613779<br>{Hemolytic uremic syndrome, atypical, susceptibility to, 5}, 612925<br>{Macular degeneration, age-related, 9}, 611378	120700	117	100	100	99

HGNC approved gene symbol	Phenotype description including OMIM phenotype ID(s)	OMIM gene ID	median depth	% covered >10x	% covered >20x	% covered 30x
C4A	[Blood group, Rodgers], 614374<br>C4a deficiency, 614380	120810	359	100	100	99
C4B	C4B deficiency, 614379	120820	352	100	100	100
C5	C5 deficiency, 609536<br>[Eculizumab, poor response to], 615749	120900	61	100	97	90
C6	C6 deficiency, 612446<br>Combined C6/C7 deficiency	217050	56	100	98	90
C7	C7 deficiency, 610102	217070	69	100	99	94
C8A	C8 deficiency, type I, 613790	120950	62	100	100	96
C8B	C8 deficiency, type II, 613789	120960	95	100	100	98
C8G	No OMIM phenotype	120930	142	100	100	100
C9	C9 deficiency, 613825<br>{Macular degeneration, age-related, 15, susceptibility to}, 615591	120940	56	100	100	93
CA2	Osteopetrosis, autosomal recessive 3, with renal tubular acidosis, 259730	611492	80	100	100	99
CARD11	B-cell expansion with NFKB and T-cell anergy, 616452<br>Immunodeficiency 11A, 615206<br>Immunodeficiency 11B with atopic dermatitis, 617638	607210	102	100	100	99
CARD14	Pityriasis rubra pilaris, 173200<br>Psoriasis 2, 602723	607211	108	100	99	98
CARD9	Candidiasis, familial, 2, autosomal recessive, 212050	607212	117	100	100	100
CARMIL2	No OMIM phenotype	610859	115	100	97	94
CASP10	Autoimmune lymphoproliferative syndrome, type II, 603909<br>Gastric cancer, somatic, 613659<br>Lymphoma, non-Hodgkin, somatic, 605027	601762	66	100	100	93
CASP8	?Autoimmune lymphoproliferative syndrome, type IIB, 607271<br>{Breast cancer, protection against}, 114480<br>Hepatocellular carcinoma, somatic, 114550<br>{Lung cancer, protection against}, 211980	601763	82	100	100	95
CAVIN1	Lipodystrophy, congenital generalized, type 4, 613327	603198	163	100	100	100
CCBE1	Hennekam lymphangiectasia-lymphedema syndrome 1, 235510	612753	71	100	98	95
CD19	Immunodeficiency, common variable, 3, 613493	107265	99	100	100	98
CD247	?Immunodeficiency 25, 610163	186780	69	100	100	98
CD27	Lymphoproliferative syndrome 2, 615122	186711	87	100	100	100
CD3D	Immunodeficiency 19, 615617	186790	85	100	100	98
CD3E	Immunodeficiency 18, 615615<br>Immunodeficiency 18, SCID variant, 615615	186830	104	100	100	100
CD3G	Immunodeficiency 17, CD3 gamma deficient, 615607	186740	66	100	100	94

HGNC approved gene symbol	Phenotype description including OMIM phenotype ID(s)	OMIM gene ID	median depth	% covered >10x	% covered >20x	% covered 30x
CD40	Immunodeficiency with hyper-IgM, type 3, 606843	109535	97	100	100	100
CD40LG	Immunodeficiency, X-linked, with hyper-IgM, 308230	300386	59	100	100	97
CD46	{Hemolytic uremic syndrome, atypical, susceptibility to, 2}, 612922	120920	79	100	99	91
CD55	[Blood group Cromer], 613793<br>Complement hyperactivation, angiopathic thrombosis, and protein-losing enteropathy, 226300	125240	48	90	83	77
CD59	Hemolytic anemia, CD59-mediated, with or without immune-mediated polyneuropathy, 612300	107271	74	100	100	99
CD70	No OMIM phenotype	602840	87	100	100	98
CD79A	Agammaglobulinemia 3, 613501	112205	78	100	95	87
CD79B	Agammaglobulinemia 6, 612692	147245	141	100	100	100
CD81	Immunodeficiency, common variable, 6, 613496	186845	140	97	90	90
CD8A	CD8 deficiency, familial, 608957	186910	104	100	100	98
CDCA7	Immunodeficiency-centromeric instability-facial anomalies syndrome 3, 616910	609937	55	100	98	88
CDKN2B	No OMIM phenotype	600431	94	100	100	100
CEBPE	Specific granule deficiency, 245480	600749	54	100	100	98
CFB	?Complement factor B deficiency, 615561<br>{Hemolytic uremic syndrome, atypical, susceptibility to, 4}, 612924<br>{Macular degeneration, age-related, 14, reduced risk of}, 615489	138470	94	100	100	100
CFD	Complement factor D deficiency, 613912	134350	96	100	100	93
CFH	Basal laminar drusen, 126700<br>Complement factor H deficiency, 609814<br>{Hemolytic uremic syndrome, atypical, susceptibility to, 1}, 235400<br>{Macular degeneration, age-related, 4}, 610698	134370	65	100	99	91
CFHR1	{Hemolytic uremic syndrome, atypical, susceptibility to}, 235400<br>{Macular degeneration, age-related, reduced risk of}, 603075	134371	48	100	93	75
CFHR3	{Hemolytic uremic syndrome, atypical, susceptibility to}, 235400<br>{Macular degeneration, age-related, reduced risk of}, 603075	605336	39	100	89	75
CFHR5	Nephropathy due to CFHR5 deficiency, 614809	608593	63	100	99	92
CFI	Complement factor I deficiency, 610984<br>{Hemolytic uremic syndrome, atypical, susceptibility to, 3}, 612923<br>{Macular degeneration, age-related, 13, susceptibility to}, 615439	217030	59	100	98	89
CFP	Properdin deficiency, X-linked, 312060	300383	63	100	94	84
CFTR	{Bronchiectasis with or without elevated sweat chloride 1, modifier of}, 211400<br>Congenital bilateral absence of vas deferens, 277180<br>Cystic fibrosis, 219700<br>{Hypertrypsinemia, neonatal}<br>{Pancreatitis, hereditary}, 167800<br>Sweat chloride elevation without CF	602421	80	100	99	93

HGNC approved gene symbol	Phenotype description including OMIM phenotype ID(s)	OMIM gene ID	median depth	% covered >10x	% covered >20x	% covered 30x
CHD7	CHARGE syndrome, 214800<br>Hypogonadotropic hypogonadism 5 with or without anosmia, 612370	608892	74	100	99	94
CIITA	Bare lymphocyte syndrome, type II, complementation group A, 209920<br>{Rheumatoid arthritis, susceptibility to}, 180300	600005	111	100	100	99
CLCN7	Osteopetrosis, autosomal dominant 2, 166600<br>Osteopetrosis, autosomal recessive 4, 611490	602727	119	100	100	99
CLEC4D	No OMIM phenotype	609964	45	100	100	87
CLEC7A	{Aspergillosis, susceptibility to}, 614079<br>Candidiasis, familial, 4, autosomal recessive, 613108	606264	51	100	98	82
CLPB	3-methylglutaconic aciduria, type VII, with cataracts, neurologic involvement and neutropenia, 616271	616254	110	100	100	99
COPA	{Autoimmune interstitial lung, joint, and kidney disease}, 616414	601924	62	100	100	96
CORO1A	Immunodeficiency 8, 615401	605000	136	100	100	99
CR2	Immunodeficiency, common variable, 7, 614699<br>{Systemic lupus erythematosus, susceptibility to, 9}, 610927	120650	52	100	100	93
CREBBP	Rubinstein-Taybi syndrome 1, 180849	600140	82	100	98	93
CSF2RA	Surfactant metabolism dysfunction, pulmonary, 4, 300770	306250	52	92	85	70
CSF2RB	Surfactant metabolism dysfunction, pulmonary, 5, 614370	138981	126	100	100	100
CSF3R	Neutropenia, severe congenital, 7, autosomal recessive, 617014	138971	96	100	100	100
CTC1	Cerebroretinal microangiopathy with calcifications and cysts, 612199	613129	90	100	100	99
CTLA4	Autoimmune lymphoproliferative syndrome, type V, 616100<br>{Celiac disease, susceptibility to, 3}, 609755<br>{Diabetes mellitus, insulin-dependent, 12}, 601388<br>{Hashimoto thyroiditis}, 140300<br>{Systemic lupus erythematosus, susceptibility to}, 152700	123890	80	100	100	99
CTPS1	Immunodeficiency 24, 615897	123860	58	100	99	92
CTSC	Haim-Munk syndrome, 245010<br>Papillon-Lefevre syndrome, 245000<br>Periodontitis 1, juvenile, 170650	602365	64	100	100	94
CXCR4	Myelokathexis, isolated<br>WHIM syndrome, 193670	162643	77	83	83	83
CYBA	Chronic granulomatous disease, autosomal, due to deficiency of CYBA, 233690	608508	116	99	99	91
CYBB	Chronic granulomatous disease, X-linked, 306400<br>Immunodeficiency 34, mycobacteriosis, X-linked, 300645	300481	43	100	97	83
DCLRE1B	No OMIM phenotype	609683	80	100	100	96
DCLRE1C	Omenn syndrome, 603554<br>Severe combined immunodeficiency, Athabascan type, 602450	605988	54	100	99	87
DDX58	Singleton-Merten syndrome 2, 616298	609631	58	100	99	91

HGNC approved gene symbol	Phenotype description including OMIM phenotype ID(s)	OMIM gene ID	median depth	% covered >10x	% covered >20x	% covered 30x
DGAT1	?Diarrhea 7, 615863	604900	108	97	91	88
DHFR	Megaloblastic anemia due to dihydrofolate reductase deficiency, 613839	126060	74	100	100	97
DKC1	Dyskeratosis congenita, X-linked, 305000	300126	43	100	97	79
DNAJC21	Bone marrow failure syndrome 3, 617052	617048	51	100	94	84
DNASE1	{Systemic lupus erythematosus, susceptibility to}, 152700	125505	124	100	100	100
DNMT3B	Immunodeficiency-centromeric instability-facial anomalies syndrome 1, 242860	602900	105	100	100	97
DOCK2	Immunodeficiency 40, 616433	603122	70	100	99	95
DOCK8	Hyper-IgE recurrent infection syndrome, autosomal recessive, 243700	611432	64	100	98	91
EFL1	Shwachman-Diamond syndrome 2, 617941	617538	54	99	96	89
ELANE	Neutropenia, cyclic, 162800<br>Neutropenia, severe congenital 1, autosomal dominant, 202700	130130	125	100	100	100
ELF4	No OMIM phenotype	300775	57	100	100	98
EPG5	Vici syndrome, 242840	615068	61	100	99	93
ERCC2	?Cerebrooculofacioskeletal syndrome 2, 610756<br>Trichothiodystrophy 1, photosensitive, 601675<br>Xeroderma pigmentosum, group D, 278730	126340	91	100	99	97
ERCC3	Trichothiodystrophy 2, photosensitive, 616390<br>Xeroderma pigmentosum, group B, 610651	133510	70	100	100	96
ERCC6L2	Bone marrow failure syndrome 2, 615715	615667	50	100	97	88
EXTL3	Immunoskeletal dysplasia with neurodevelopmental abnormalities, 617425	605744	133	100	100	100
F12	Angioedema, hereditary, type III, 610618<br>Factor XII deficiency, 234000	610619	134	100	100	99
FAAP24	No OMIM phenotype	610884	98	100	100	100
FADD	Infections, recurrent, with encephalopathy, hepatic dysfunction, and cardiovascular malformations, 613759	602457	131	100	100	100
FAS	Autoimmune lymphoproliferative syndrome, type IA, 601859<br>{Autoimmune lymphoproliferative syndrome}, 601859<br>Squamous cell carcinoma, burn scar-related, somatic	134637	239	100	99	90
FASLG	Autoimmune lymphoproliferative syndrome, type IB, 601859<br>{Lung cancer, susceptibility to}, 211980	134638	51	100	100	97
FAT4	Hennekam lymphangiectasia-lymphedema syndrome 2, 616006<br>Van Maldergem syndrome 2, 615546	612411	78	100	100	98
FCGR1A	[IgG receptor I, phagocytic, familial deficiency of]	146760	99	100	100	99
FCGR2A	{Lupus nephritis, susceptibility to}, 152700<br>{Malaria, severe, susceptibility to}, 611162<br>{Pseudomonas aeruginosa, susceptibility to chronic infection by, in cystic fibrosis}, 219700	146790	136	100	100	100
FCGR2B	{Malaria, resistance to}, 611162<br>{Systemic lupus erythematosus, susceptibility to}, 152700	604590	219	100	100	100

HGNC approved gene symbol	Phenotype description including OMIM phenotype ID(s)	OMIM gene ID	median depth	% covered >10x	% covered >20x	% covered 30x
FCGR3A	Immunodeficiency 20, 615707	146740	248	100	100	97
FCGR3B	Neutropenia, alloimmune neonatal	610665	217	100	100	100
FCN3	Immunodeficiency due to ficolin 3 deficiency, 613860	604973	91	100	100	95
FERMT3	Leukocyte adhesion deficiency, type III, 612840	607901	109	100	100	95
FOXN1	T-cell immunodeficiency, congenital alopecia, and nail dystrophy, 601705	600838	119	100	100	100
FOXP3	{Diabetes mellitus, type I, susceptibility to}, 222100<br>Immunodysregulation, polyendocrinopathy, and enteropathy, X-linked, 304790	300292	60	100	99	90
FPR1	No OMIM phenotype	136537	102	100	100	100
G6PC	Glycogen storage disease Ia, 232200	613742	96	100	100	100
G6PC3	Dursun syndrome, 612541<br>Neutropenia, severe congenital 4, autosomal recessive, 612541	611045	96	100	100	100
G6PD	Hemolytic anemia, G6PD deficient (favism), 300908<br>{Resistance to malaria due to G6PD deficiency}, 611162	305900	90	100	100	99
GATA2	Emberger syndrome, 614038<br>Immunodeficiency 21, 614172<br>{Leukemia, acute myeloid, susceptibility to}, 601626<br>{Myelodysplastic syndrome, susceptibility to}, 614286	137295	97	100	100	100
GFI1	?Neutropenia, nonimmune chronic idiopathic, of adults, 607847<br>?Neutropenia, severe congenital 2, autosomal dominant, 613107	600871	134	100	100	100
GINS1	Immunodeficiency 55, 617827	610608	59	100	99	84
GJC2	Leukodystrophy, hypomyelinating, 2, 608804<br>Lymphedema, hereditary, IC, 613480<br>Spastic paraplegia 44, autosomal recessive, 613206	608803	92	98	88	81
GRHL2	Corneal dystrophy, posterior polymorphous, 4, 618031<br>Deafness, autosomal dominant 28, 608641<br>Ectodermal dysplasia/short stature syndrome, 616029	608576	69	100	100	96
GTF2H5	Trichothiodystrophy 3, photosensitive, 616395	608780	44	100	100	90
HAX1	Neutropenia, severe congenital 3, autosomal recessive, 610738	605998	90	100	100	98
HELLS	Immunodeficiency-centromeric instability-facial anomalies syndrome 4, 616911	603946	54	100	93	78
HMOX1	Heme oxygenase-1 deficiency, 614034<br>{Pulmonary disease, chronic obstructive, susceptibility to}, 606963	141250	102	100	100	100
HYOU1	No OMIM phenotype	601746	100	100	100	97
ICOS	Immunodeficiency, common variable, 1, 607594	604558	49	100	99	90
IFIH1	Aicardi-Goutieres syndrome 7, 615846<br>Singleton-Merten syndrome 1, 182250	606951	74	100	99	93
IFNAR2	{Hepatitis B virus, susceptibility to}, 610424<br>?Immunodeficiency 45, 616669	602376	79	100	100	93

HGNC approved gene symbol	Phenotype description including OMIM phenotype ID(s)	OMIM gene ID	median depth	% covered >10x	% covered >20x	% covered 30x
IFNGR1	{H. pylori infection, susceptibility to}, 600263<br>{Hepatitis B virus infection, susceptibility to}, 610424<br>Immunodeficiency 27A, mycobacteriosis, AR, 209950<br>Immunodeficiency 27B, mycobacteriosis, AD, 615978<br>{Tuberculosis infection, protection against}, 607948<br>{Tuberculosis, susceptibility to}, 607948	107470	61	100	99	90
IFNGR2	Immunodeficiency 28, mycobacteriosis, 614889	147569	53	97	92	86
IGLL1	Agammaglobulinemia 2, 613500	146770	127	100	100	100
IKBKB	Immunodeficiency 15, 615592	603258	79	100	99	95
IKBKG	Ectodermal dysplasia, hypohidrotic, with immune deficiency, 300291<br>Ectodermal, dysplasia, anhidrotic, lymphedema and immunodeficiency, 300301<br>Immunodeficiency 33, 300636<br>Immunodeficiency, isolated, 300584<br>Incontinentia pigmenti, 308300<br>Invasive pneumococcal disease, recurrent isolated, 2, 300640	300248	77	100	100	96
IKZF1	Immunodeficiency, common variable, 13, 616873	603023	140	100	100	99
IL10	{Graft-versus-host disease, protection against}, 614395<br>{HIV-1, susceptibility to}, 609423<br>{Rheumatoid arthritis, progression of}, 180300	124092	114	100	100	96
IL10RA	Inflammatory bowel disease 28, early onset, autosomal recessive, 613148	146933	119	100	100	99
IL10RB	{Hepatitis B virus, susceptibility to}, 610424<br>Inflammatory bowel disease 25, early onset, autosomal recessive, 612567	123889	60	100	99	93
IL12B	Immunodeficiency 29, mycobacteriosis, 614890	161561	68	100	100	92
IL12RB1	Immunodeficiency 30, 614891	601604	105	100	100	99
IL17F	?Candidiasis, familial, 6, autosomal dominant, 613956	606496	72	100	100	100
IL17RA	Immunodeficiency 51, 613953	605461	114	100	100	100
IL17RC	Candidiasis, familial, 9, 616445	610925	106	100	100	99
IL1RN	{Gastric cancer risk after H. pylori infection}, 137215<br>Interleukin 1 receptor antagonist deficiency, 612852<br>{Microvascular complications of diabetes 4}, 612628	147679	72	100	99	86
IL2	No OMIM phenotype	147680	35	100	85	59
IL21	?Immunodeficiency, common variable, 11, 615767	605384	56	100	97	86
IL21R	[IgE, elevated level of], 147050<br>Immunodeficiency 56, 615207	605383	104	100	100	100
IL2RA	{Diabetes, mellitus, insulin-dependent, susceptibility to, 10}, 601942<br>Immunodeficiency 41 with lymphoproliferation and autoimmunity, 606367	147730	89	100	100	99

HGNC approved gene symbol	Phenotype description including OMIM phenotype ID(s)	OMIM gene ID	median depth	% covered >10x	% covered >20x	% covered 30x
IL2RG	Combined immunodeficiency, X-linked, moderate, 312863<br>Severe combined immunodeficiency, X-linked, 300400	308380	49	100	99	81
IL36RN	Psoriasis 14, pustular, 614204	605507	79	100	100	100
IL7R	Severe combined immunodeficiency, T-cell negative, B-cell/natural killer cell-positive type, 608971	146661	52	100	100	94
INO80	No OMIM phenotype	610169	65	100	97	85
INSR	Diabetes mellitus, insulin-resistant, with acanthosis nigricans, 610549<br>Hyperinsulinemic hypoglycemia, familial, 5, 609968<br>Leprechaunism, 246200<br>Rabson-Mendenhall syndrome, 262190	147670	102	100	98	95
IRAK1	No OMIM phenotype	300283	63	100	99	91
IRAK4	IRAK4 deficiency, 607676<br>Invasive pneumococcal disease, recurrent isolated, 1, 610799	606883	63	100	98	86
IRF2BP2	?Immunodeficiency, common variable, 14, 617765	615332	85	100	100	99
IRF3	{Encephalopathy, acute, infection-induced (herpes-specific), susceptibility to, 7}, 616532	603734	121	100	99	96
IRF7	?Immunodeficiency 39, 616345	605047	123	100	99	97
IRF8	Immunodeficiency 32A, mycobacteriosis, autosomal dominant, 614893<br>Immunodeficiency 32B, monocyte and dendritic cell deficiency, autosomal recessive, 226990	601565	135	100	100	99
ISG15	Immunodeficiency 38, 616126	147571	135	100	100	100
ITCH	Autoimmune disease, multisystem, with facial dysmorphism, 613385	606409	52	96	94	86
ITGB2	Leukocyte adhesion deficiency, 116920	600065	142	100	100	100
ITK	Lymphoproliferative syndrome 1, 613011	186973	69	100	100	92
JAGN1	Neutropenia, severe congenital, 6, autosomal recessive, 616022	616012	75	100	100	100
JAK1	No OMIM phenotype	147795	60	100	97	89
JAK2	{Budd-Chiari syndrome, somatic}, 600880<br>Erythrocytosis, somatic, 133100<br>Leukemia, acute myeloid, somatic, 601626<br>Myelofibrosis, somatic, 254450<br>Polycythemia vera, somatic, 263300<br>Thrombocythemia 3, 614521	147796	54	100	97	85
JAK3	SCID, autosomal recessive, T-negative/B-positive type, 600802	600173	99	100	100	99
KDM6A	Kabuki syndrome 2, 300867	300128	43	100	95	72
KMT2A	Leukemia, myeloid/lymphoid or mixed-lineage, 159555<br>Wiedemann-Steiner syndrome, 605130	159555	60	100	100	95
KMT2D	Kabuki syndrome 1, 147920	602113	106	100	100	99
LAMTOR2	Immunodeficiency due to defect in MAPBP-interacting protein, 610798	610389	96	100	100	100
LAT	Immunodeficiency 52, 617514	602354	84	100	100	97
LCK	?Immunodeficiency 22, 615758	153390	155	100	100	100

HGNC approved gene symbol	Phenotype description including OMIM phenotype ID(s)	OMIM gene ID	median depth	% covered >10x	% covered >20x	% covered 30x
LIG1	No OMIM phenotype	126391	86	100	100	98
LIG4	LIG4 syndrome, 606593<br>{Multiple myeloma, resistance to}, 254500	601837	61	100	100	100
LPIN2	Majeed syndrome, 609628	605519	65	100	99	93
LRBA	Immunodeficiency, common variable, 8, with autoimmunity, 614700	606453	55	100	97	88
LRRC8A	?Agammaglobulinemia 5, 613506	608360	152	100	100	100
LTBP3	Dental anomalies and short stature, 601216<br>Geleophysic dysplasia 3, 617809	602090	125	100	99	96
LYST	Chediak-Higashi syndrome, 214500	606897	62	100	98	91
MAGT1	Immunodeficiency, X-linked, with magnesium defect, Epstein-Barr virus infection and neoplasia, 300853	300715	44	100	97	81
MAL	No OMIM phenotype	188860	92	100	100	93
MALT1	Immunodeficiency 12, 615468	604860	57	97	93	83
MAN2B1	Mannosidosis, alpha-, types I and II, 248500	609458	110	100	100	100
MANBA	Mannosidosis, beta, 248510	609489	76	100	98	92
MAP3K14	No OMIM phenotype	604655	109	100	100	100
MASP2	MASP2 deficiency, 613791	605102	80	100	99	94
MBL2	{Chronic infections, due to MBL deficiency}, 614372	154545	122	100	100	100
MC2R	Glucocorticoid deficiency, due to ACTH unresponsiveness, 202200	607397	87	100	100	100
MCM4	Immunodeficiency 54, 609981	602638	79	100	100	95
MEFV	Familial Mediterranean fever, AD, 134610<br>Familial Mediterranean fever, AR, 249100	608107	99	100	100	100
MKL1	Megakaryoblastic leukemia, acute	606078	92	98	96	91
MOGS	Congenital disorder of glycosylation, type IIb, 606056	601336	117	100	100	100
MRE11	Ataxia-telangiectasia-like disorder 1, 604391	600814	47	100	94	74
MS4A1	Immunodeficiency, common variable, 5, 613495	112210	72	100	100	96
MSN	Immunodeficiency 50, 300988	309845	44	98	91	75
MTHFD1	Combined immunodeficiency and megaloblastic anemia with or without hyperhomocysteinemia, 617780<br>{Neural tube defects, folate-sensitive, susceptibility to}, 601634	172460	67	100	99	91
MVK	Hyper-IgD syndrome, 260920<br>Mevalonic aciduria, 610377<br>Porokeratosis 3, multiple types, 175900	251170	96	100	100	98
MYD88	Macroglobulinemia, Waldenstrom, somatic, 153600<br>Pyogenic bacterial infections, recurrent, due to MYD88 deficiency, 612260	602170	140	100	100	100
MYSM1	No OMIM phenotype	612176	47	100	96	81
NBAS	Infantile liver failure syndrome 2, 616483<br>Short stature, optic nerve atrophy, and Pelger-Huet anomaly, 614800	608025	57	100	98	89

HGNC approved gene symbol	Phenotype description including OMIM phenotype ID(s)	OMIM gene ID	median depth	% covered >10x	% covered >20x	% covered 30x
NBN	Aplastic anemia, 609135<br>Leukemia, acute lymphoblastic, 613065<br>Nijmegen breakage syndrome, 251260	602667	59	100	97	81
NCF1	Chronic granulomatous disease due to deficiency of NCF-1, 233700	608512	174	100	100	99
NCF2	Chronic granulomatous disease due to deficiency of NCF-2, 233710	608515	84	100	100	96
NCF4	?Granulomatous disease, chronic, autosomal recessive, cytochrome b-positive, type III, 613960	601488	110	100	100	100
NCSTN	Acne inversa, familial, 1, 142690	605254	89	100	100	98
NFAT5	No OMIM phenotype	604708	54	100	98	92
NFKB1	Immunodeficiency, common variable, 12, 616576	164011	48	100	96	84
NFKB2	Immunodeficiency, common variable, 10, 615577	164012	136	100	100	94
NFKBIA	Ectodermal dysplasia, anhidrotic, with T-cell immunodeficiency, 612132	164008	134	100	100	100
NHEJ1	Severe combined immunodeficiency with microcephaly, growth retardation, and sensitivity to ionizing radiation, 611291	611290	47	100	100	89
NHP2	Dyskeratosis congenita, autosomal recessive 2, 613987	606470	102	100	100	100
NKX2-5	Atrial septal defect 7, with or without AV conduction defects, 108900<br>Conotruncal heart malformations, variable, 217095<br>Hypoplastic left heart syndrome 2, 614435<br>Hypothyroidism, congenital nongoitrous, 5, 225250<br>Tetralogy of Fallot, 187500<br>Ventricular septal defect 3, 614432	600584	106	100	100	98
NLRC4	Autoinflammation with infantile enterocolitis, 616050<br>?Familial cold autoinflammatory syndrome 4, 616115	606831	67	100	100	96
NLRP1	Autoinflammation with arthritis and dyskeratosis, 617388<br>Palmoplantar carcinoma, multiple self-healing, 615225<br>{Vitiligo-associated multiple autoimmune disease susceptibility 1}, 606579	606636	96	100	100	99
NLRP12	Familial cold autoinflammatory syndrome 2, 611762	609648	127	100	100	100
NLRP3	CINCA syndrome, 607115<br>Deafness, autosomal dominant 34, with or without inflammation, 617772<br>Familial cold inflammatory syndrome 1, 120100<br>Keratoendothelitis fugax hereditaria, 148200<br>Muckle-Wells syndrome, 191900	606416	108	100	100	100
NOD2	Blau syndrome, 186580<br>{Inflammatory bowel disease 1, Crohn disease}, 266600<br>{Psoriatic arthritis, susceptibility to}, 607507<br>{Yao syndrome}, 617321	605956	104	100	100	97
NOP10	Dyskeratosis congenita, autosomal recessive 1, 224230	606471	133	100	100	100

HGNC approved gene symbol	Phenotype description including OMIM phenotype ID(s)	OMIM gene ID	median depth	% covered >10x	% covered >20x	% covered 30x
NRAS	Colorectal cancer, somatic, 114500<br>Epidermal nevus, somatic, 162900<br>Melanocytic nevus syndrome, congenital, somatic, 137550<br>Neurocutaneous melanosis, somatic, 249400<br>Noonan syndrome 6, 613224<br>?RAS-associated autoimmune lymphoproliferative syndrome type IV, somatic, 614470<br>Schimmelpenning-Feuerstein-Mims syndrome, somatic mosaic, 163200<br>Thyroid carcinoma, follicular, somatic, 188470	164790	53	100	100	95
NSMCE3	Lung disease, immunodeficiency, and chromosome breakage syndrome, 617241	608243	150	100	100	100
ORAI1	Immunodeficiency 9, 612782<br>Myopathy, tubular aggregate, 2, 615883	610277	155	100	99	97
OSTM1	Osteopetrosis, autosomal recessive 5, 259720	607649	69	100	99	92
OTULIN	Autoinflammation, panniculitis, and dermatosis syndrome, 617099	615712	64	98	93	85
PARN	Dyskeratosis congenita, autosomal recessive 6, 616353<br>Pulmonary fibrosis and/or bone marrow failure, telomere-related, 4, 616371	604212	52	100	99	88
PAX5	{Leukemia, acute lymphoblastic, susceptibility to, 3}, 615545	167414	99	100	99	93
PBX1	Congenital anomalies of kidney and urinary tract syndrome with or without hearing loss, abnormal ears, or developmental delay, 617641	176310	65	100	100	95
PCCA	Propionicacidemia, 606054	232000	58	100	99	90
PCCB	Propionicacidemia, 606054	232050	62	100	97	92
PEPD	Prolidase deficiency, 170100	613230	92	100	100	97
PGM3	Immunodeficiency 23, 615816	172100	75	100	100	94
PIGA	Multiple congenital anomalies-hypotonia-seizures syndrome 2, 300868<br>Paroxysmal nocturnal hemoglobinuria, somatic, 300818	311770	56	100	100	93
PIK3CD	Immunodeficiency 14, 615513	602839	127	100	99	98
PIK3R1	?Agammaglobulinemia 7, autosomal recessive, 615214<br>Immunodeficiency 36, 616005<br>SHORT syndrome, 269880	171833	72	100	100	94
PLCG2	Autoinflammation, antibody deficiency, and immune dysregulation syndrome, 614878<br>Familial cold autoinflammatory syndrome 3, 614468	600220	101	100	100	97
PLEKHM1	Osteopetrosis, autosomal recessive 6, 611497	611466	181	100	100	100
PLG	Dysplasminogenemia, 217090<br>Plasminogen deficiency, type I, 217090	173350	73	100	100	97
PMM2	Congenital disorder of glycosylation, type Ia, 212065	601785	66	100	100	93
PNP	Immunodeficiency due to purine nucleoside phosphorylase deficiency, 613179	164050	61	100	99	94
POLA1	Pigmentary disorder, reticulate, with systemic manifestations, X-linked, 301220	312040	38	99	89	70
POLE2	No OMIM phenotype	602670	37	99	88	61

HGNC approved gene symbol	Phenotype description including OMIM phenotype ID(s)	OMIM gene ID	median depth	% covered >10x	% covered >20x	% covered 30x
POT1	{Glioma susceptibility 9}, 616568<br>{Melanoma, cutaneous malignant, susceptibility to, 10}, 615848	606478	59	100	99	89
PRF1	Aplastic anemia, 609135<br>Hemophagocytic lymphohistiocytosis, familial, 2, 603553<br>Lymphoma, non-Hodgkin, 605027	170280	114	100	100	100
PRKCD	Autoimmune lymphoproliferative syndrome, type III, 615559	176977	108	100	100	100
PRKDC	Immunodeficiency 26, with or without neurologic abnormalities, 615966	600899	65	100	98	89
PRPS1	Arts syndrome, 301835<br>Charcot-Marie-Tooth disease, X-linked recessive, 5, 311070<br>Deafness, X-linked 1, 304500, X-linked<br>Gout, PRPS-related, 300661<br>Phosphoribosylpyrophosphate synthetase superactivity, 300661	311850	44	100	96	81
PSENEN	Acne inversa, familial, 2, with or without Dowling-Degos disease, 613736	607632	68	100	100	100
PSMB8	Autoinflammation, lipodystrophy, and dermatosis syndrome, 256040	177046	119	100	100	99
PSTPIP1	Pyogenic sterile arthritis, pyoderma gangrenosum, and acne, 604416	606347	111	100	100	99
PTPN22	{Diabetes, type 1, susceptibility to}, 222100<br>{Rheumatoid arthritis, susceptibility to}, 180300<br>{Systemic lupus erythematosus susceptibility to}, 152700	600716	45	100	98	81
PTPRC	{Hepatitis C virus, susceptibility to}, 609532<br>Severe combined immunodeficiency, T cell-negative, B-cell/natural killer-cell positive, 608971	151460	64	100	98	92
RAB27A	Griscelli syndrome, type 2, 607624	603868	44	100	98	81
RAC2	Neutrophil immunodeficiency syndrome, 608203	602049	99	100	100	100
RAG1	Alpha/beta T-cell lymphopenia with gamma/delta T-cell expansion, severe cytomegalovirus infection, and autoimmunity, 609889<br>Combined cellular and humoral immune defects with granulomas, 233650<br>Omenn syndrome, 603554<br>Severe combined immunodeficiency, B cell-negative, 601457	179615	81	100	100	100
RAG2	Combined cellular and humoral immune defects with granulomas, 233650<br>Omenn syndrome, 603554<br>Severe combined immunodeficiency, B cell-negative, 601457	179616	58	100	100	98
RANBP2	{Encephalopathy, acute, infection-induced, 3, susceptibility to}, 608033	601181	109	100	100	98
RASGRP1	No OMIM phenotype	603962	62	100	100	97
RASGRP2	?Bleeding disorder, platelet-type, 18, 615888	605577	90	100	100	100
RBCK1	Polyglucosan body myopathy 1 with or without immunodeficiency, 615895	610924	132	100	100	100
RECQL4	Baller-Gerold syndrome, 218600<br>RAPADILINO syndrome, 266280<br>Rothmund-Thomson syndrome, 268400	603780	137	100	100	99

HGNC approved gene symbol	Phenotype description including OMIM phenotype ID(s)	OMIM gene ID	median depth	% covered >10x	% covered >20x	% covered 30x
RELB	?Immunodeficiency 53, 617585	604758	83	100	99	95
RFX5	Bare lymphocyte syndrome, type II, complementation group C, 209920<br>Bare lymphocyte syndrome, type II, complementation group E, 209920	601863	86	100	99	95
RFXANK	MHC class II deficiency, complementation group B, 209920	603200	110	100	100	100
RFXAP	Bare lymphocyte syndrome, type II, complementation group D, 209920	601861	139	100	100	100
RHOH	No OMIM phenotype	602037	89	100	100	100
RMRP	Anauxetic dysplasia 1, 607095<br>Cartilage-hair hypoplasia, 250250<br>Metaphyseal dysplasia without hypotrichosis, 250460	157660	No coverage	0	0	0
RNASEH2A	Aicardi-Goutieres syndrome 4, 610333	606034	98	100	100	100
RNASEH2B	Aicardi-Goutieres syndrome 2, 610181	610326	54	100	100	88
RNASEH2C	Aicardi-Goutieres syndrome 3, 610329	610330	279	100	100	100
RNF168	RIDDLE syndrome, 611943	612688	81	100	100	98
RNF31	No OMIM phenotype	612487	108	100	100	99
RNU4ATAC	Microcephalic osteodysplastic primordial dwarfism, type I, 210710<br>Roifman syndrome, 616651	601428	No coverage	0	0	0
RORC	Immunodeficiency 42, 616622	602943	91	100	100	99
RPSA	Asplenia, isolated congenital, 271400	150370	83	100	100	100
RSPH9	Ciliary dyskinesia, primary, 12, 612650	612648	121	100	100	100
RTEL1	Dyskeratosis congenita, autosomal dominant 4, 615190<br>Dyskeratosis congenita, autosomal recessive 5, 615190<br>Pulmonary fibrosis and/or bone marrow failure, telomere-related, 3, 616373	608833	132	100	100	99
SAMD9	MIRAGE syndrome, 617053<br>Tumoral calcinosis, familial, normophosphatemic, 610455	610456	59	100	100	99
SAMD9L	Ataxia-pancytopenia syndrome, 159550	611170	53	100	100	97
SAMHD1	Aicardi-Goutieres syndrome 5, 612952<br>?Chilblain lupus 2, 614415	606754	58	100	97	80
SBDS	{Aplastic anemia, susceptibility to}, 609135<br>Shwachman-Diamond syndrome, 260400	607444	72	100	100	98
SEMA3E	?CHARGE syndrome, 214800	608166	53	100	99	87
SERAC1	3-methylglutaconic aciduria with deafness, encephalopathy, and Leigh-like syndrome, 614739	614725	56	100	95	79
SERPING1	Angioedema, hereditary, types I and II, 106100<br>Complement component 4, partial deficiency of, 120790	606860	105	100	100	98
SH2B3	Erythrocytosis, somatic, 133100<br>Myelofibrosis, somatic, 254450<br>Thrombocythemia, somatic, 187950	605093	109	100	100	100
SH2D1A	Lymphoproliferative syndrome, X-linked, 1, 308240	300490	51	100	94	81

HGNC approved gene symbol	Phenotype description including OMIM phenotype ID(s)	OMIM gene ID	median depth	% covered >10x	% covered >20x	% covered 30x
SH3BP2	Cherubism, 118400	602104	113	91	91	91
SKIV2L	Trichohepatoenteric syndrome 2, 614602	600478	116	100	100	99
SLC29A3	Histiocytosis-lymphadenopathy plus syndrome, 602782	612373	139	100	99	99
SLC35A1	Congenital disorder of glycosylation, type IIf, 603585	605634	60	100	100	93
SLC35C1	Congenital disorder of glycosylation, type IIc, 266265	605881	133	100	100	100
SLC37A4	Glycogen storage disease Ib, 232220<br>Glycogen storage disease Ic, 232240	602671	87	100	99	93
SLC39A4	Acrodermatitis enteropathica, 201100	607059	122	100	100	100
SLC46A1	Folate malabsorption, hereditary, 229050	611672	113	100	100	100
SMARCAL1	Schimke immunoosseous dysplasia, 242900	606622	77	100	100	96
SMARCD2	Specific granule deficiency 2, 617475	601736	71	87	85	83
SNX10	Osteopetrosis, autosomal recessive 8, 615085	614780	65	100	100	94
SOCS4	No OMIM phenotype	616337	67	100	100	100
SP110	Hepatic venoocclusive disease with immunodeficiency, 235550<br>{Mycobacterium tuberculosis, susceptibility to}, 607948	604457	52	100	99	90
SPINK5	Netherton syndrome, 256500	605010	55	100	98	89
SRP54	No OMIM phenotype	604857	48	100	95	84
STAT1	Immunodeficiency 31A, mycobacteriosis, autosomal dominant, 614892<br>Immunodeficiency 31B, mycobacterial and viral infections, autosomal recessive, 613796<br>Immunodeficiency 31C, autosomal dominant, 614162	600555	57	100	99	91
STAT2	Immunodeficiency 44, 616636	600556	74	100	99	93
STAT3	Autoimmune disease, multisystem, infantile-onset, 1, 615952<br>Hyper-IgE recurrent infection syndrome, 147060	102582	80	100	100	96
STAT4	{Systemic lupus erythematosus, susceptibility to, 11}, 612253	600558	54	100	98	85
STAT5B	Growth hormone insensitivity with immunodeficiency, 245590<br>Leukemia, acute promyelocytic, somatic, 102578	604260	115	100	99	93
STAT6	No OMIM phenotype	601512	87	100	100	97
STIM1	Immunodeficiency 10, 612783<br>Myopathy, tubular aggregate, 1, 160565<br>Stormorken syndrome, 185070	605921	81	100	100	97
STK4	T-cell immunodeficiency, recurrent infections, autoimmunity, and cardiac malformations, 614868	604965	71	100	100	97
STN1	Cerebroretinal microangiopathy with calcifications and cysts 2, 617341	613128	56	100	97	84
STX11	Hemophagocytic lymphohistiocytosis, familial, 4, 603552	605014	268	100	100	100
STXBP2	Hemophagocytic lymphohistiocytosis, familial, 5, 613101	601717	109	100	100	99
TAP1	Bare lymphocyte syndrome, type I, 604571	170260	119	100	100	100
TAP2	Bare lymphocyte syndrome, type I, due to TAP2 deficiency, 604571<br>Wegener-like granulomatosis	170261	99	100	100	100

HGNC approved gene symbol	Phenotype description including OMIM phenotype ID(s)	OMIM gene ID	median depth	% covered >10x	% covered >20x	% covered 30x
TAPBP	Bare lymphocyte syndrome, type I, 604571	601962	102	100	100	100
TAZ	Barth syndrome, 302060	300394	89	100	97	90
TBX1	Conotruncal anomaly face syndrome, 217095<br>DiGeorge syndrome, 188400<br>Tetralogy of Fallot, 187500<br>Velocardiofacial syndrome, 192430	602054	81	94	85	79
TCF3	Agammaglobulinemia 8, autosomal dominant, 616941	147141	84	100	100	98
TCIRG1	Osteopetrosis, autosomal recessive 1, 259700	604592	116	100	100	99
TCN2	Transcobalamin II deficiency, 275350	613441	107	100	100	100
TERC	{Aplastic anemia}, 614743<br>Dyskeratosis congenita, autosomal dominant 1, 127550<br>{Pulmonary fibrosis, idiopathic, susceptibility to}, 614743	602322	No coverage	0	0	0
TERT	{Dyskeratosis congenita, autosomal dominant 2}, 613989<br>{Dyskeratosis congenita, autosomal recessive 4}, 613989<br>{Leukemia, acute myeloid}, 601626<br>{Melanoma, cutaneous malignant, 9}, 615134<br>{Pulmonary fibrosis and/or bone marrow failure, telomere-related, 1}, 614742	187270	131	100	100	99
TFRC	Immunodeficiency 46, 616740	190010	50	100	98	82
THBD	{Hemolytic uremic syndrome, atypical, susceptibility to, 6}, 612926<br>Thrombophilia due to thrombomodulin defect, 614486	188040	199	100	100	100
TICAM1	{Encephalopathy, acute, infection-induced (herpes-specific), susceptibility to, 6}, 614850	607601	131	100	100	97
TINF2	Dyskeratosis congenita, autosomal dominant 3, 613990<br>Revesz syndrome, 268130	604319	140	100	100	100
TIRAP	{Bacteremia, protection against}, 614382<br>{Malaria, protection against}, 611162<br>{Pneumococcal disease, invasive, protection against}, 610799<br>{Tuberculosis, protection against}, 607948	606252	155	100	100	100
TLR3	{Encephalopathy, acute, infection-induced (herpes-specific), susceptibility to, 2}, 613002<br>{HIV1 infection, resistance to}, 609423	603029	56	100	100	97
TLR4	No OMIM phenotype	603030	55	100	100	97
TMC6	Epidermodysplasia verruciformis, 226400	605828	84	100	100	98
TMC8	Epidermodysplasia verruciformis, 226400	605829	111	100	100	100
TMEM173	STING-associated vasculopathy, infantile-onset, 615934	612374	116	100	100	100
TNFAIP3	Autoinflammatory syndrome, familial, Behcet-like, 616744	191163	96	100	99	95
TNFRSF11A	Osteolysis, familial expansile, 174810<br>Osteopetrosis, autosomal recessive 7, 612301<br>{Paget disease of bone 2, early-onset}, 602080	603499	90	95	95	95

HGNC approved gene symbol	Phenotype description including OMIM phenotype ID(s)	OMIM gene ID	median depth	% covered >10x	% covered >20x	% covered 30x
TNFRSF13B	Immunodeficiency, common variable, 2, 240500<br>Immunoglobulin A deficiency 2, 609529	604907	89	100	100	100
TNFRSF13C	Immunodeficiency, common variable, 4, 613494	606269	70	100	98	80
TNFRSF1A	{Multiple sclerosis, susceptibility to, 5}, 614810<br>Periodic fever, familial, 142680	191190	85	100	97	94
TNFRSF4	?Immunodeficiency 16, 615593	600315	67	99	91	84
TNFSF11	Osteopetrosis, autosomal recessive 2, 259710	602642	47	100	99	84
TNFSF12	No OMIM phenotype	602695	71	100	99	95
TPP2	No OMIM phenotype	190470	49	100	97	84
TRAF3	{?Encephalopathy, acute, infection-induced (herpes-specific), susceptibility to, 5}, 614849	601896	100	100	100	99
TRAF3IP2	?Candidiasis, familial, 8, 615527<br>{Psoriasis susceptibility 13}, 614070	607043	91	100	100	98
TREX1	Aicardi-Goutieres syndrome 1, dominant and recessive, 225750<br>Chilblain lupus, 610448<br>{Systemic lupus erythematosus, susceptibility to}, 152700<br>Vasculopathy, retinal, with cerebral leukodystrophy, 192315	606609	217	100	100	100
TRNT1	Retinitis pigmentosa and erythrocytic microcytosis, 616959<br>Sideroblastic anemia with B-cell immunodeficiency, periodic fevers, and developmental delay, 616084	612907	58	100	98	87
TTC37	Trichohepatoenteric syndrome 1, 222470	614589	50	100	98	87
TTC7A	Gastrointestinal defects and immunodeficiency syndrome, 243150	609332	109	100	100	100
TYK2	Immunodeficiency 35, 611521	176941	123	100	100	99
UNC13D	Hemophagocytic lymphohistiocytosis, familial, 3, 608898	608897	96	100	100	100
UNC93B1	{Encephalopathy, acute, infection-induced (herpes-specific), susceptibility to, 1}, 610551	608204	73	98	90	82
UNG	Immunodeficiency with hyper IgM, type 5, 608106	191525	104	100	100	100
USB1	Poikiloderma with neutropenia, 604173	613276	90	100	100	97
USP18	Pseudo-TORCH syndrome 2, 617397	607057	120	100	95	95
VAV1	No OMIM phenotype	164875	85	100	99	92
VPS13B	Cohen syndrome, 216550	607817	63	100	98	91
VPS45	Neutropenia, severe congenital, 5, autosomal recessive, 615285	610035	64	100	100	92
WAS	Neutropenia, severe congenital, X-linked, 300299<br>Thrombocytopenia, X-linked, 313900<br>Thrombocytopenia, X-linked, intermittent, 313900<br>Wiskott-Aldrich syndrome, 301000	300392	62	95	81	75
WDR1	No OMIM phenotype	604734	71	100	98	93
WIPF1	?Wiskott-Aldrich syndrome 2, 614493	602357	71	100	99	94
WRAP53	Dyskeratosis congenita, autosomal recessive 3, 613988	612661	144	100	100	100
XIAP	Lymphoproliferative syndrome, X-linked, 2, 300635	300079	39	100	93	78

HGNC approved gene symbol	Phenotype description including OMIM phenotype ID(s)	OMIM gene ID	median depth	% covered >10x	% covered >20x	% covered 30x
ZAP70	Autoimmune disease, multisystem, infantile-onset, 2, 617006<br>Immunodeficiency 48, 269840	176947	149	100	100	100
ZBTB24	Immunodeficiency-centromeric instability-facial anomalies syndrome 2, 614069	614064	66	100	100	98

- Gene symbols according HGCN
- OMIM release used: 4-7-2018
- "No OMIM phenotypes" indicates a gene without a current OMIM association
- OMIM phenotypes between "[ ]", indicate "nondiseases," mainly genetic variations that lead to apparently abnormal laboratory test values
- OMIM phenotypes between "{}", indicate risk factors
- OMIM phenotypes with a question mark, "?", before the disease name indicates an unconfirmed or possibly spurious mapping
- The statistics above are based on a set of 95 samples
- Median depth is the median of the mean sequence depth over the protein coding exons (±10bp flanking introns) of the longest transcript
- % Covered 10x describes the percentage of a gene’s coding sequence (±10bp flanking introns) that is covered at least 10x
- % Covered 20x describes the percentage of a gene’s coding sequence (±10bp flanking introns) that is covered at least 20x
- % Covered 30x describes the percentage of a gene’s coding sequence (±10bp flanking introns) that is covered at least 30x
